# EPIC: multi-objective guided diffusion for epitope design in TCR-pMHC complexes

**DOI:** 10.1093/bioinformatics/btag358

**Published:** 2026-06-04

**Authors:** Yueshan Huang, Gufeng Yu, Letian Chen, Haoyang Luan, Yang Yang

**Affiliations:** AGI Institute, School of Computer Science, Shanghai Jiao Tong University, Shanghai 200240, China; AGI Institute, School of Computer Science, Shanghai Jiao Tong University, Shanghai 200240, China; AGI Institute, School of Computer Science, Shanghai Jiao Tong University, Shanghai 200240, China; Shanghai Innovation Institute, Shanghai 200231, China; AGI Institute, School of Computer Science, Shanghai Jiao Tong University, Shanghai 200240, China; AGI Institute, School of Computer Science, Shanghai Jiao Tong University, Shanghai 200240, China

## Abstract

**Motivation:**

T cell receptor (TCR) recognition of peptide-major histocompatibility complex (pMHC) complexes is central to adaptive immunity, yet rational design of immunogenic epitopes remains elusive due to complex triplet binding constraints and data scarcity. No existing method can generate epitopes satisfying simultaneous requirements for antigenicity, MHC presentation, and TCR specificity.

**Results:**

We present EPIC, a multi-objective diffusion framework that decomposes TCR-pMHC binding into three biologically grounded sub-tasks, enabling training-free gradient guidance without end-to-end retraining. By integrating ESM-based classifiers with a peptide diffusion generator, EPIC leverages heterogeneous immunological interaction datasets to generate diverse, context-aware epitopes. EPIC-designed top-three epitopes achieve lower predicted interface energies compared to ground-truth epitopes in 78.31% of test cases, while maintaining 80.1% sequence novelty and comparable structural confidence. Generated epitopes exhibit 100% uniqueness, high diversity (64.05%), and high antigenicity scores (0.4723). To our knowledge, EPIC is the first computational framework capable of *de novo* epitope design while explicitly integrating the triplet constraints of TCR-pMHC binding. This paradigm shift from discovery to design unlocks new potential for personalized cancer vaccines, precision adoptive T cell therapy, and rapid response to emerging infectious diseases.

**Availability and implementation:**

The source code of EPIC is available at https://github.com/Octopus125/EPIC and archived on Zenodo (DOI: 10.5281/zenodo.18537646).

## 1 Introduction

Specific recognition between T cell receptors (TCRs) and peptides presented by major histocompatibility complex (MHC) molecules plays a central role in adaptive immunity. This exquisitely specific molecular interaction has become a focal point for cancer immunotherapy development, particularly following the clinical success of adoptive T cell therapies (Cowell 2020, Kidman *et al.* 2020, Valpione *et al.* 2021). Recent advances in adoptive T cell therapies, such as CAR-T and TCR-engineered T cells, have achieved remarkable clinical success in hematological malignancies (Sterner and Sterner 2021, Vincent *et al.* 2023). However, extending these therapies to solid tumors, which account for over 90% of cancer deaths, remains challenging due to the scarcity of tumor-specific antigens and the difficulty in identifying immunogenic epitopes that can be effectively presented and recognized (Baulu *et al.* 2023). Complementary to engineering the TCR itself, rational design of the target epitopes offers an alternative approach to enhance therapeutic efficacy. Optimized epitope sequences can boost immune recognition, improve MHC presentation stability, and reduce off-target effects (Musunuri *et al.* 2024), thereby addressing the antigen scarcity problem from the opposite direction. Consequently, the design of epitopes that can be efficiently presented by MHC molecules and precisely recognized by specific TCRs has attracted increasing attention in recent years (Duperret *et al.* 2019, Gigoux *et al.* 2022, Liu *et al.* 2024b).

However, addressing the full complexity of TCR-pMHC interactions remains a challenge for current computational approaches. While general multi-objective peptide generation frameworks (Rector-Brooks *et al.* 2025, Chen *et al.* 2025b,c, Tang *et al.* 2025a,b) have demonstrated remarkable success in optimizing scalar properties, such as solubility and antimicrobial activity, extending these methods to the TCR-pMHC context is hindered by the lack of sufficiently accurate and robust computational proxies, where no reliable scalar metric currently exists to accurately capture the complex, cooperative protein-protein interactions (PPIs). On the other hand, generative methods tailored for the TCR-pMHC domain consider only a single constraint, focusing on either the TCR or MHC component in isolation. For instance, EpiGen (Ma *et al.* 2025) generates epitopes conditioned on TCR CDR3 *β* sequences without MHC constraints, whereas PepPPO (Chen *et al.* 2023) optimizes MHC presentation for specific alleles while ignoring TCR recognition. Such designs fail to capture the cooperative and interdependent nature of TCR-MHC-peptide interactions, where antigenicity, MHC presentation, and TCR recognition must be satisfied simultaneously. In addition, existing methods for analyzing TCR-antigen specificity typically focus only on the CDR3 *β* of the TCR, overlooking the contributions of other CDR loops and framework regions that also participate in the structural interplay between TCR and epitope (Zhang *et al.* 2024). Consequently, no existing method performs *de novo* epitope design that simultaneously satisfies the complex constraints imposed by both TCR and MHC recognition and captures the full molecular context of immune recognition.

This methodological gap is fundamentally rooted in severe data scarcity. Only ∼1000 experimentally validated TCR-pMHC triplets with complete sequence information are publicly available (Zhang *et al.* 2024, Zhao *et al.* 2025), compared to ∼10 000 antibody-antigen structures (Dunbar *et al.* 2014) and >300 000 MHC-peptide pairs (Albert *et al.* 2023). This scarcity, reflecting the experimental difficulty of cellular TCR-pMHC assays, makes end-to-end deep learning on triplet data impractical. Moreover, available datasets suffer from (i) extreme class imbalance, with <1% positive binding rates in experimental screens (Deng *et al.* 2023); and (ii) incomplete full-length paired sequence data, as most provide only TCR CDR3 *β* sequences while omitting *α*-chains and framework regions. These compounding challenges of scarce, imbalanced, and incomplete triplet data explain why rational *de novo* epitope design for TCR-pMHC complexes remains an open challenge.

**Figure 1:**
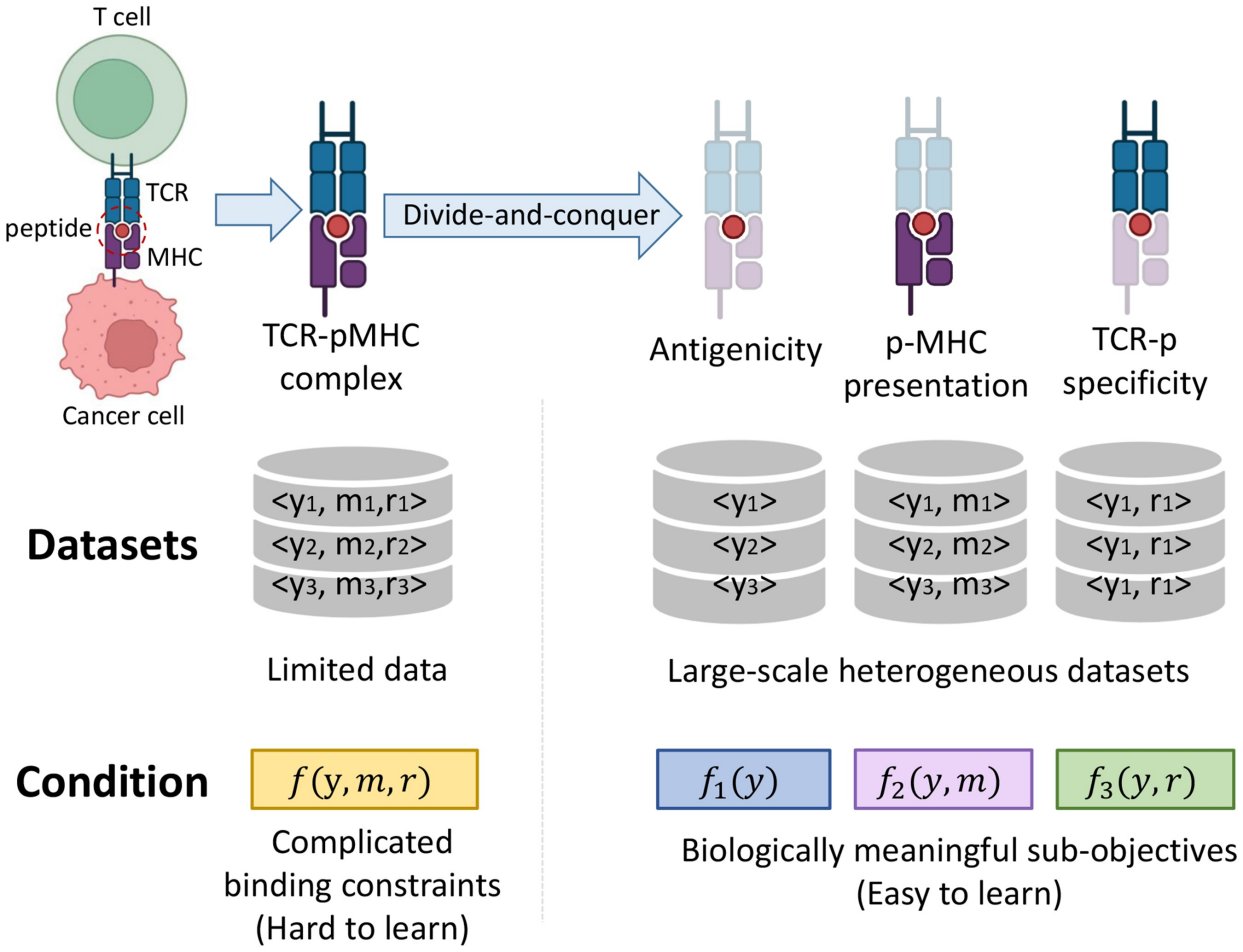
A divide-and-conquer strategy for modeling TCR-pMHC binding constraints, which enables simplified modeling and improved data utilization.

Here we present EPIC (EPItope design via multi-objective guided diffusion for TCR-pMHC Complexes), the first computational framework capable of *de novo* epitope design under full TCR-pMHC constraints. EPIC introduces three key innovations:

- *Biologically grounded decomposition*: We reformulate the triplet binding problem into three biologically meaningful sub-objectives: antigenicity, MHC presentation, and TCR recognition (Fig. 1). This decomposition simplifies constraint modeling and enables effective use of heterogeneous datasets.
- *Context-aware multi-objective guidance*: EPIC integrates ESM-based classifiers with a diffusion generator through training-free gradient guidance, enabling flexible constraint control and scalability without model retraining.
- *Large-scale heterogeneous dataset integration*: We construct large-scale, heterogeneous datasets containing paired full-length TCR sequences, enabling EPIC to achieve strong generalization under limited or imbalanced triplet data.

EPIC represents a paradigm shift from passive epitope discovery to active, rational design, with immediate applications in neoantigen vaccine development, TCR-T cell therapy optimization, and autoimmune disease treatment. The source code of EPIC is available at https://github.com/Octopus125/EPIC and archived on Zenodo (DOI: 10.5281/zenodo.18537646).

## 2 Materials and methods

### 2.1 Problem definition and formulation

The objective of this study is to design epitope sequences that can be effectively presented by a given MHC and specifically recognized by a target TCR, thereby forming a stable TCR-pMHC complex. Let *r* and *m* denote the full-length amino acid sequences of a TCR and an MHC, respectively. Let y∈Y denote a peptide sequence of max length *L*, where each residue $y_{i}$ is a one-hot vector over the standard amino acid vocabulary A={A,C,D,…,Y}.

*Task definition.* The goal of this problem is to find epitope sequences that can form a stable TCR-pMHC complex given specific TCR and MHC sequences. This is formally expressed as a constraint satisfaction problem:


(1) {y∈Y|f(y,m,r)=1},

where f(y,m,r) is an indicator of whether y,m,r can form a stable TCR-pMHC complex.

### 2.2 Model architecture

#### 2.2.1 Overview of EPIC framework

**Figure 2:**
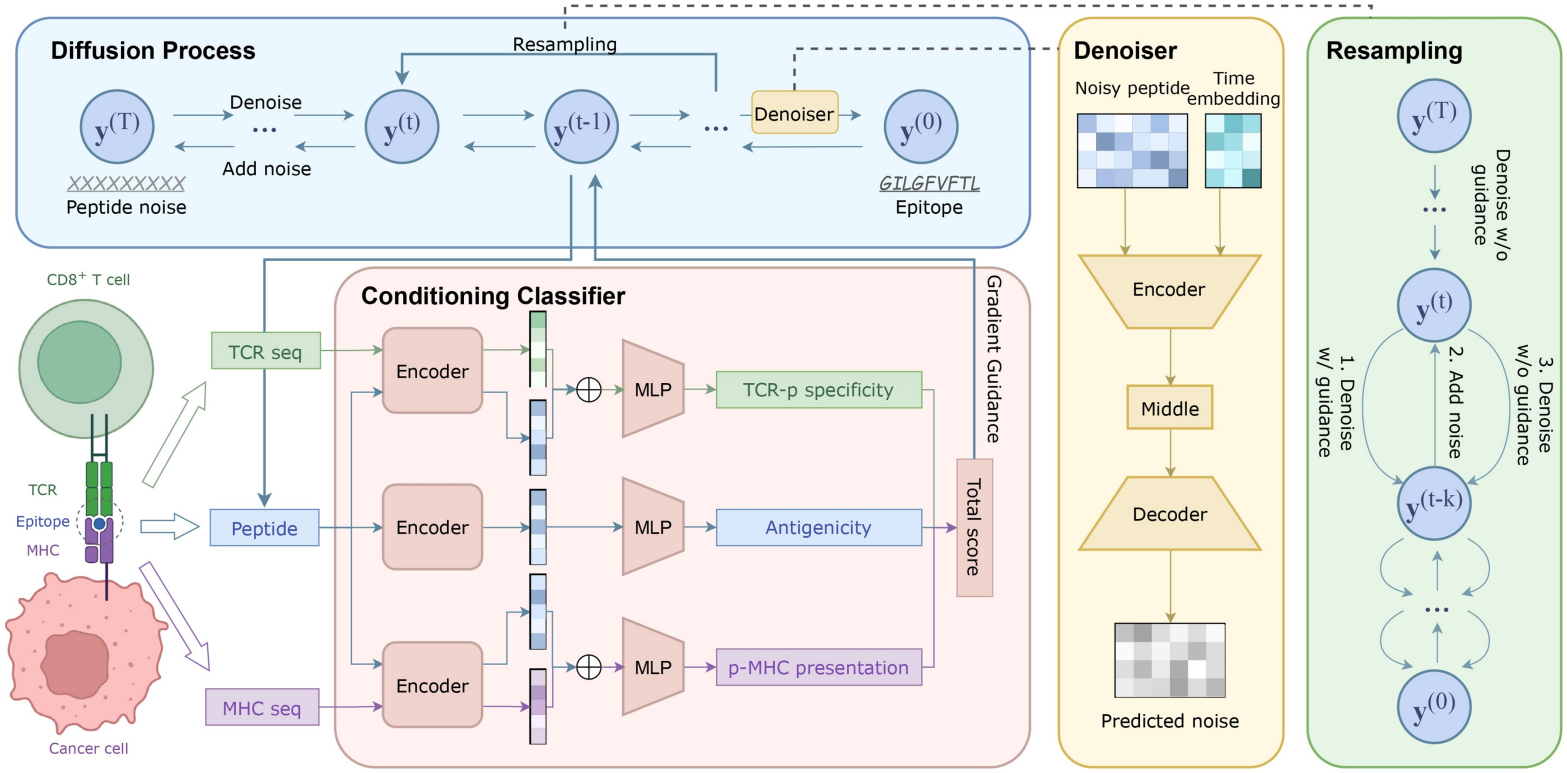
Overall architecture of EPIC.

As shown in Fig. 2, our proposed model, EPIC, comprises two main components: a peptide generator based on score-based diffusion (Song *et al.* 2021), and a set of discriminative classifiers that evaluate the immunological validity of generated sequences. The generator is responsible for producing peptide candidates in an unconditional manner through a diffusion process, while the classifiers provide conditional signals to control the generation process through training-free gradient guidance. The classifier module evaluates generated peptides based on three aspects: antigenicity, MHC presentation, and TCR specificity.

We transform the constraint satisfaction problem in Equation (1) into an optimization problem:


(2) $$\arg\underset{y\in Y}{\max}p(c_{A}=1,c_{T}=1,c_{M}=1|y,m,r),$$

where $c_{T}$ represents TCR-p specificity, $c_{M}$ represents p-MHC presentation, and $c_{A}$ is a conditioning variable indicating the desired antigenic property that the generated peptide *y* should possess. Directly solving Equation (2) can be computationally intractable due to the vast search space and the complexity of the joint probability function. Therefore, this problem is reformulated as a relaxed problem, specifically a conditional generation task. Instead of directly searching for the single optimal sequence, the objective becomes sampling epitope sequences from a parametrized probability model: (In the subsequent discussion of this paper, unless otherwise specified, the values of the variables $c_{A}$, $c_{T}$, and $c_{M}$ are set to 1, i.e., $c_{A}=1$, $c_{T}=1$, and $c_{M}=1$.)


(3) $$y∼p_{\theta }(\cdot |c_{A},c_{T},c_{M},r,m),\;y\in Y.$$

This parametrized probability model aims to approximate the true conditional probability distribution $p(y|c_{A},c_{T},c_{M},r,m)$.

#### 2.2.2 Peptide generator

We adopt a continuous-time diffusion framework, specifically Score-Based Generative Modeling (SGM) using Stochastic Differential Equations (SDEs) (Song *et al.* 2021), to model the generation of peptide sequences. This approach operates directly in the one-hot encoded peptide space.

*Diffusion process.* The forward diffusion process gradually perturbs a clean peptide sequence y(0) into a standard Gaussian noise vector y(T) over a continuous time interval t∈[0,T]. This is described by the following SDE:


(4) dy=f(y,t)dt+g(t)dw,

where w is a standard Wiener process, f(y,t) is the drift coefficient, and g(t) is the diffusion coefficient.

The generative process involves reversing this diffusion. Starting from a sample y(T)∼N(0,I), we solve the corresponding reverse-time SDE from t=T to t=0:


(5) $$dy=\left[f(y,t)-g{\left(t\right)}^{2}s_{\theta }(y,t)\right]dt+g(t)d\bar{w},$$

where $d\bar{w}$ is a standard Wiener process when time flows from *T* to 0 (i.e., in reverse), and $s_{\theta }(y,t)$ denotes the learned score function, implemented as a time-dependent neural network trained to approximate a score function $\nabla_{y(t)}\;\text{log}\;p_{t}(y(t))$, with $p_{t}(y(t))$ being the marginal probability density of y(t). This SDE (Equation 5) is solved numerically by sampling y(T)∼N(0,I) and integrating backwards in time to t=0.

*Network architecture.* The backbone of the scoring function $s_{\theta }(y(t),t)$ is a 1D U-Net. This architecture captures local residue patterns using convolutional layers and skip connections. Given that peptides are typically short (8–13 residues), local context is often sufficient to model biologically relevant features, making convolutional architectures a natural and efficient choice.

#### 2.2.3 Classifiers for guided generation

To guide the generative process with TCR-pMHC binding constraints, we employ a suite of three pre-trained classifiers, denoted ${\left\{f_{i}\right\}}_{i=1}^{3}$. Each classifier is designed to assess a distinct dimension of biological relevance. All classifiers share a common architectural design: they utilize the ESM model (Lin *et al.* 2023) as a sequence encoder, followed by a multi-layer perceptron (MLP) head for binary classification. The individual classifiers are defined as follows:

1. *Antigenicity Classifier* ($f_{1}(y)$): This classifier predicts the general antigenicity of a peptide *y*. It models the conditional probability $p(c_{A}|y)$.
2. *pMHC Presentation Classifier* ($f_{2}(y,m)$): Given a peptide *y* and a specific MHC *m*, this classifier predicts the probability that *y* can be presented by *m*. It models the conditional probability $p(c_{M}|y,m)$.
3. *TCR-Peptide Specificity Classifier* ($f_{3}(y,r)$): For a peptide *y* and a specific TCR *r*, this classifier evaluates the probability that *y* is recognized by *r*. It models the conditional probability $p(c_{T}|y,r)$.

Each classifier $f_{i}$ outputs a scalar value in the range [0,1], representing the probability of satisfying the respective biological constraint ($c_{A}$, $c_{M}$, or $c_{T}$). The overall objective is to estimate the joint probability of these three constraints being simultaneously satisfied, given the peptide *y* and the specific molecular contexts *m* and *r*, i.e., $p(c_{A},c_{M},c_{T}|y,m,r)$. Assuming conditional independence of these constraints (specifically, we assume $c_{A}$ is independent of *m* and *r* given *y*; $c_{M}$ is independent of *r* given *y* and *m*; and $c_{T}$ is independent of *m* given *y* and *r*), this joint probability can be factorized and approximated as follows:


(6) $$\begin{array}{cc} p(c_{A},c_{M},c_{T}|y,m,r) & =p(c_{A}|y)p(c_{M}|y,m)p(c_{T}|y,r)\\ & \approx f_{1}(y)f_{2}(y,m)f_{3}(y,r). \end{array}$$

We denote this function as g(y,m,r) for further use.

Detailed information about the model implementation can be found in Supplementary Information S1.1, available as supplementary data at *Bioinformatics* online.

### 2.4 Modular training

Our model is trained using a modular training paradigm. The diffusion-based generator and the discriminative classifiers are trained independently. Moreover, each predictor module within the classifier is also trained separately. Detailed training procedures are provided in Supplementary Information S1.2, available as supplementary data at *Bioinformatics* online.

Modular training is motivated by the scarcity of comprehensive triplet data for the TCR-pMHC complex (Zhang *et al.* 2024, Zhao *et al.* 2025). Existing datasets are small, biased, and lack peptide diversity, making end-to-end training of generative models challenging. Modular training allows EPIC to utilize available biological datasets effectively, bypassing the need for fully labeled triplets. This approach improves training efficiency and convergence, enhances model generalization through diverse, high-quality datasets, and enables flexible adaptation to new tasks without retraining, ensuring scalability and adaptability to changing data landscapes.

### 2.5 Sampling strategy

The inference phase of EPIC follows the standard sampling procedure in a score-based SDEs. Since our generator only models the probability distribution of peptide $p_{\theta }(y)$, gradient-guided sampling is introduced to transform the peptide distribution $p_{\theta }(y)$ to the conditional probability distribution $p(y|c_{A},c_{T},c_{M},r,m)$. We also leverage the resampling mechanism to increase the sampling quality.

*Gradient-guided sampling.* Based on Bayes’ rule, we can estimate the conditional probability distribution $p(y|c_{A},c_{T},c_{M},r,m)$ as follows:


(7) $$\begin{array}{cc} p(y|c_{A},c_{M},c_{T},m,r) & \propto p(c_{A},c_{M},c_{T}|y,m,r)p(y)\\ & \approx g(y,m,r)p_{\theta }(y), \end{array}$$

where Equation (7) derives from Equation (6). In order to model the conditional probability distribution $p(y|c_{A},c_{M},c_{T},m,r)$, the score function in Equation (5) can be modified as:


(8) $$\begin{array}{c} s_{\theta }(y,t)=\nabla_{y(t)}\;\text{log}\;p_{t}(y(t)|c_{A},c_{M},c_{T},m,r)\\ =\nabla_{y(t)}\;\text{log}\;p_{t}(y(t))+\nabla_{y(t)}\;\text{log}\;p_{t}(c_{A},c_{M},c_{T}|y(t),m,r)\\ \approx \nabla_{y(t)}\;\text{log}\;p_{t}(y(t))+\nabla_{{\hat{y}}_{0}}\;\text{log}\;p(c_{A},c_{M},c_{T}|{\hat{y}}_{0},m,r)\\ \approx \nabla_{y(t)}\;\text{log}\;p_{t}(y(t))+\eta \nabla_{{\hat{y}}_{0}}\;\text{log}\;g({\hat{y}}_{0},m,r), \end{array}$$

where ${\hat{y}}_{0}$ is the one-step estimation from $y_{t}$ using the learned scoring function, and η is a hyperparameter used to control the gradient scale. The estimation in the second line of Equation (8) is proved in (Han *et al.* 2023). In this manner, the scoring function for the target distribution is estimated by the linear combination of the score provided by the scoring network and the gradient from the pre-trained classifiers, with no additional training process involved.

*Resampling strategy.* To further improve generation quality, we implement a resampling mechanism (Lugmayr *et al.* 2022, Ye *et al.* 2024) to improve the sampled peptides toward the desired properties. Despite the usefulness of classifier guidance, excessive reliance on gradient signals may lead to unrealistic or invalid peptide sequences. In particular, the classifiers, trained on limited datasets, inevitably capture some noise and struggle to fully model the complex patterns underlying TCR-pMHC recognition. To mitigate this problem, we propose a *hybrid guidance resampling* strategy, in which we interleave gradient-guided and unguided denoising steps within the resampling process. More details are shown in Supplementary Information S1.3, available as supplementary data at *Bioinformatics* online. This hybrid guidance resampling strategy prevents overfitting to potentially noisy classifier gradients. By periodically reverting to unconditional denoising, the model maintains generative diversity and ensures peptide validity in the physical and biological sense.

### 2.6 Datasets

We construct three datasets to support training and evaluation. For TCR-pMHC triplet data, we use the BEAM-T dataset from 10x Genomics (https://www.10xgenomics.com/library/a14cde) (10x Genomics 2022), which contains paired and full-length TCR sequences, MHC alleles, peptide sequences, and their specificity scores. After pre-processing and deduplication, we obtain 953 positive and 15 793 negative triplets. This dataset is primarily used to train the TCR-p specificity classifier and to evaluate the final generation performance of our model. For antigenicity modeling, we curate a labeled peptide dataset from IEDB (Vita *et al.* 2019), which contains 8793 positive and 17 401 negative samples after removing duplicates. Positive samples are also used to train the unconditional diffusion generator. To model MHC presentation, we use the dataset in BigMHC (Albert *et al.* 2023), which includes 333 437 positive and 17 639 877 negative peptide-MHC pairs. Given the class imbalance across these datasets, we employ stratified sampling to split the data into training and test sets at a 9:1 ratio. Detailed information about the datasets can be found in Supplementary Information S2.1, available as supplementary data at *Bioinformatics* online.

### 2.7 Comparison with baseline methods

To the best of our knowledge, no existing computational method has been specifically designed for epitope generation based on TCR and MHC sequences. As baselines, we evaluate several large language models (LLMs), including ChatGPT-4o (Islam and Moushi 2024), DeepSeek-v3 (Liu *et al.* 2024a), and Gemini-2.5-flash (Team *et al.* 2023), as potential peptide generators. Additionally, we further evaluate several peptide generation methods relevant to this task, including EpiGen (Ma *et al.* 2025), PepPPO (Chen *et al.* 2023), PepTune (Tang *et al.* 2025a), MOG-DFM (Chen *et al.* 2025c), PepMLM (Chen *et al.* 2025a), and DPLM (Wang *et al.* 2024). EpiGen generates epitopes based solely on the CDR3 *β* regions of the TCR, while PepPPO relies exclusively on the MHC allele to optimize peptide sequences through reinforcement learning. PepTune and MOG-DFM are state-of-the-art multi-objective optimization frameworks for peptide design. Since these methods primarily target scalar property optimization, we employ the outputs of our classifiers as their optimization rewards. PepMLM is a general framework for designing potential peptide binders for target proteins, and DPLM is a protein design model that supports sequence-conditioned generation, both of which can jointly accept TCR and MHC sequences as input to perform epitope generation. For reference, we also generate a set of random sequences based on the residue distribution observed in the peptides within the dataset. Detailed information about the baselines can be found in Supplementary Information S2.2, available as supplementary data at *Bioinformatics* online.

### 2.8 Evaluation metrics

We evaluate all methods on 83 test cases, with each method generating 100 peptide candidates per case, resulting in a total of 8300 peptides generated. Some key metrics are used for comparison: (i) *Similarity*, which represents how closely the generated sequences match existing ones, with a lower score indicating higher novelty; (ii) *Diversity*, which measures the variety of the generated sequences, with a higher score indicating a broader range of unique sequences; (iii) *Uniqueness*, which denotes the percentage of unique peptides generated, reflecting the model’s ability to produce distinct sequences; (iv) *plDDT*${\;}^{p}$, which quantifies the confidence in the predicted local structure for each residue of the generated peptide; and (v) *Antigenicity score*, computed using MHCflurry (O’Donnell *et al.* 2020), which reflects the predicted probability that a peptide can be effectively presented by the target MHC, serving as an indicator of its potential antigenicity. Detailed information about the metrics can be found in Supplementary Information S2.3, available as supplementary data at *Bioinformatics* online.

## 3 Experimental results

### 3.1 EPIC achieves superior performance across sequence, structure, and functional metrics

Table 1 summarizes the performance of EPIC and baseline methods across several key evaluation metrics. More detailed experimental results are shown in Supplementary Information S3.1, S3.3, and S3.4, available as supplementary data at *Bioinformatics* online.

**Table 1:** Performance comparison between EPIC and baseline methods on the peptide design task.

Methods	Similarity (%)↓	Diversity (%)↑	Uniqueness (%)↑	plDDT${\;}^{p}$ ↑	Antigenicity score ↑
Random sampling	26.77	74.94	100.00	0.6837	0.0628
ChatGPT 4o (zero-shot)	37.52	58.39	52.01	0.6527	0.1971
ChatGPT 4o (few-shot)	32.23	63.09	44.23	0.7256	0.3257
DeepSeek-v3 (zero-shot)	27.00	61.53	47.67	0.6322	0.1435
DeepSeek-v3 (few-shot)	38.93	47.28	28.98	0.7160	0.1987
Gemini-2.5-flash (zero-shot)	32.02	62.92	93.65	0.7303	0.3847
Gemini-2.5-flash (few-shot)	38.46	56.91	71.60	0.7343	0.4301
EpiGen	47.26	48.14	48.35	0.6483	0.0617
PepPPO	29.11	66.80	99.98	0.7008	**0.8628**
PepTune	23.44	64.05	**100.00**	0.6989	0.0283
MOG-DFM	22.74	71.28	**100.00**	0.6869	0.0212
PepMLM	**14.63**	43.02	99.59	0.7276	0.0177
DPLM	19.82	**72.53**	94.82	0.7047	0.0409
EPIC (unconditional)	28.13	71.88	**100.00**	0.7182	0.2486
EPIC (conditional)	28.27	64.05	**100.00**	**0.7443**	0.4723

The best and second-best results in each column are shown in bold and underlined, respectively. The shaded row (Random sampling) is provided only as a reference baseline rather than a generative method, and is therefore excluded from the best/second-best ranking. ↑ indicates that a higher value is better; ↓ indicates that a lower value is better.

Overall, EPIC exhibits strong and balanced performance across all dimensions of evaluation. Specifically, at the sequence level (similarity, diversity, and uniqueness), it produces highly diverse and non-redundant peptides, achieving the highest uniqueness and competitive diversity and similarity scores among all models. This indicates that our method excels at generating diverse and novel sequences without producing repetitive or highly similar peptides to those found in the real world. At the structure level, EPIC also achieves the highest plDDT${\;}^{p}$ value (0.7443), suggesting that the generated peptides are more likely to form spatially coherent and biophysically compatible local conformations within the modeled complex and therefore to form stable TCR-pMHC assemblies. Meanwhile, EPIC attains high antigenicity scores (0.4723), second only to PepPPO, which explicitly uses MHCflurry2.0 as its optimization reward. The fact that EPIC achieves comparable results without direct optimization against the same predictor underscores its genuine biological generalization and its capacity to produce peptides that are both immunologically relevant and structurally feasible. Together, these findings indicate that EPIC effectively balances novelty, diversity, and biological constraint satisfaction, enabling the generation of epitopes that are both innovative and functionally credible.

In contrast, the baseline methods exhibit various limitations, each constrained by their modeling assumptions or lack of domain-specific integration. For LLM-based methods, while few-shot prompting improves their structure-level metrics and functional performance, they often produce repetitive and similar peptides. When evaluating plDDT${\;}^{p}$, we observe that the instances where LLMs perform well often match the ground truth sequences, while other results are significantly inferior. This suggests that in some specific scientific domains, LLMs have limited reasoning capabilities, making it challenging for them to uncover novel domain knowledge. EpiGen, which relies solely on the TCR CDR3β region, significantly underperforms compared to other methods, especially in MHC-related metrics (plDDT${\;}^{p}$ and antigenicity score). This highlights the importance of fully utilizing the full-length protein sequences and incorporating multiple biological constraints in the TCR-pMHC complex modeling and design. PepPPO achieves outstanding results on antigenicity (0.8628), consistent with its reinforcement learning reward directly derived from MHCflurry2.0 predictions. However, as it does not incorporate TCR information, its only moderate structural correctness (plDDT${\;}^{p}$ = 0.7008) suggests that it fails to generate peptides capable of forming structurally coherent TCR-pMHC complexes. Although the multi-objective optimization methods PepTune and MOG-DFM achieve perfect uniqueness and competitive diversity, they exhibit low antigenicity scores and moderate structural confidence. This performance gap primarily stems from their reliance on external scoring functions, which provide coarse-grained guidance that struggles to navigate the fitness landscape of cooperative PPIs without residue-level gradients. Furthermore, the strict Pareto optimization strategies of PepTune limit exploration efficiency in the tightly coupled objective space, often retaining early, mediocre solutions. PepMLM produces highly novel peptides but suffers from low diversity and almost negligible antigenicity, reflecting its limited capability in learning biologically grounded constraints. DPLM, as a general protein design model, performs well on sequence-level metrics but exhibits a low antigenicity score, suggesting that generic protein inpainting is insufficient to capture the immunological specificity required for epitope design.

Collectively, these results demonstrate that EPIC not only generates diverse and novel peptide sequences but also achieves superior structural and functional validity. By integrating multi-objective biological constraints, EPIC effectively captures the principles underlying TCR-pMHC recognition, yielding epitopes with high specificity, plausible structure, and strong potential for immune activation.

### 3.2 EPIC generates structurally plausible epitopes with lower predicted interface energies

**Figure 3:**
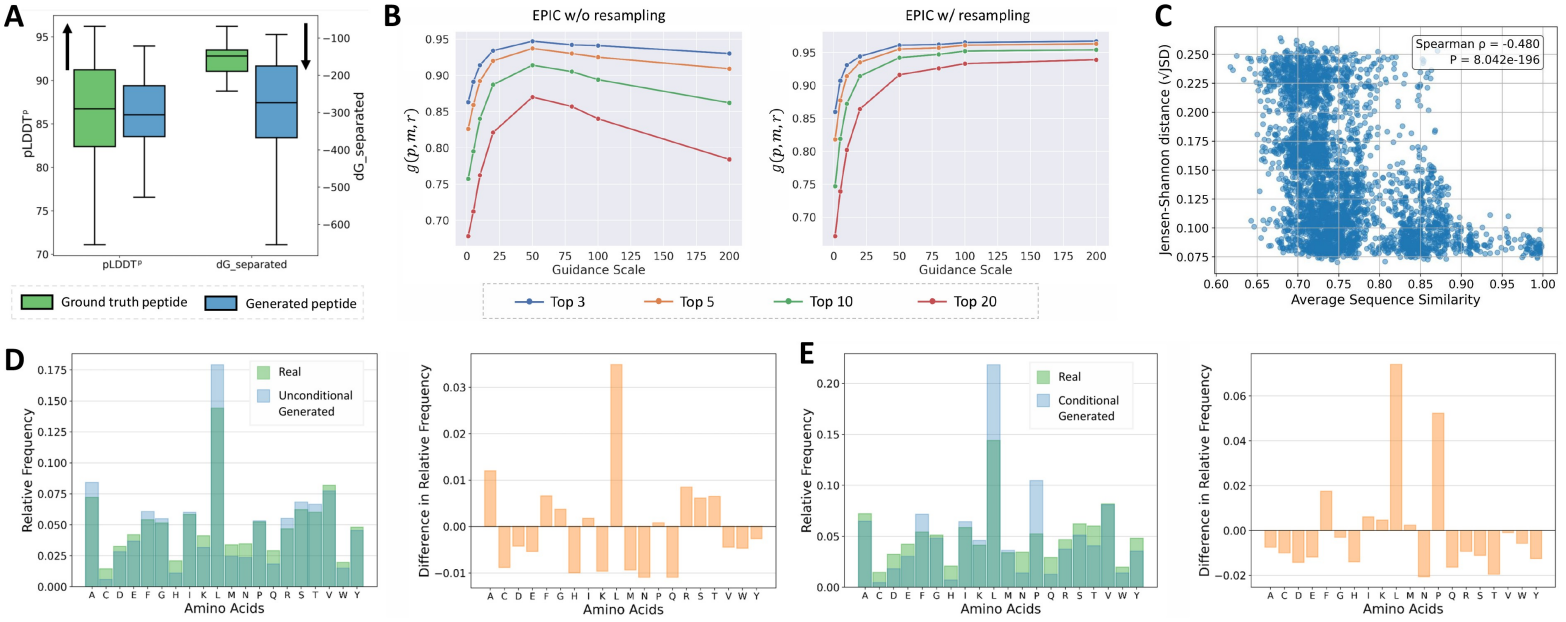
(A) Box plot of predicted peptide structural confidence (plDDT${\;}^{p}$) and binding affinity (dG_separated) for EPIC top 3 versus ground-truth epitopes. (B) Ablation study on impact of guidance scale and resampling strategy. (C) Scatter plot of the mean position-wise Jensen-Shannon distance (${\bar{D}}_{JS}$) versus TCR-MHC sequence similarity, reflecting context-dependent adaptation of EPIC-generated peptide distributions. (D) Amino acid distributions of peptides generated by EPIC without guidance compared with native epitopes. (E) Amino acid distributions of peptides generated by EPIC with guidance compared with native epitopes.

To further demonstrate the effectiveness of EPIC, we use AlphaFold 3 to predict the structures of TCR-pMHC complexes for the top-three designed peptides for each TCR target. We analyze the plDDT${\;}^{p}$ of the peptide regions and evaluate predicted binding energetics using Rosetta InterfaceAnalyzer (Stranges and Kuhlman 2013). As shown in Fig. 3A, EPIC-generated peptides exhibit significantly lower predicted interface energies (mean dG_separated = −175.42 Rosetta Energy Units (REU)) compared to the reference epitopes, outperforming the ground-truth peptides in 78.31% of cases. This substantial reduction in calculated energy suggests that the designed epitopes possess a higher potential for strong binding affinity and complex stability. Complementing this energetic analysis, we examine the AlphaFold 3 confidence scores as a measure of structural plausibility. The plDDT${\;}^{p}$ values of generated peptides are comparable to those of experimentally validated epitopes, with 44.6% of cases achieving higher plDDT${\;}^{p}$ than the reference sequences while maintaining a sequence novelty of 80.1%. This convergence of low interface energy and high model confidence strongly supports the biological validity of the designs.

**Figure 4:**
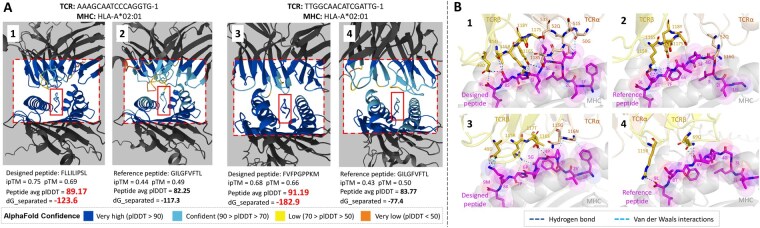
(A) AlphaFold3-predicted structures and model confidence for two representative TCR-pMHC complexes. Each case compares a designed peptide (left) with the experimentally validated reference peptide (right). (B) Visualization of the corresponding core interaction regions.

Figure 4 shows the structural predictions for two representative cases, where the designed peptides are compared with experimentally validated reference epitopes. These two TCRs are both common TCR variants and play a crucial role in immune activation (Brownlie and Zamoyska 2013, Rossjohn *et al.* 2015). Interface interaction analyses reveal that EPIC-designed peptides form additional hydrogen bonds and van der Waals contacts with multiple CDR loops, particularly CDR3 regions, enhancing interface complementarity and recognition specificity. These optimized contacts directly contribute to the lower predicted interface energies observed. Consistent with this favorable energetic profile, the designed peptides achieve higher plDDT scores than the reference epitopes, indicating more confident structural predictions of the peptide conformations. Furthermore, we observe enhanced plDDT confidence in the MHC and the CDR loops of the TCR near the binding interface. Together, these results demonstrate that EPIC can generate peptides with both lower interface energies and higher structural plausibility, potentially surpassing known epitopes in predicted structural and energetic stability. Additional examples are provided in Supplementary Information S3.2, available as supplementary data at *Bioinformatics* online.

### 3.3 Gradient-guided generation enables precise multi-objective control

We conduct ablation studies to investigate two key aspects in EPIC: the influence of classifier guidance strength and resampling strategy on generation quality, and the individual contributions of each constraint-specific classifier module.

*Impact of guidance scale and resampling.* The strength of classifier guidance during sampling is controlled by the guidance scale η. As shown in Fig. 3B, we evaluate the generation quality under different guidance scales with and without the resampling mechanism. The quality of generated peptides is directly measured by the joint probability g(p,m,r) mentioned in Equation (6).

For the model without the resampling mechanism, we observe that performance improves as the guidance scale increases initially, but then deteriorates when the guidance becomes too strong. This suggests that overly strong guidance can distort the generative prior, leading to unrealistic or over-optimized peptides that no longer resemble valid biological sequences. In contrast, when resampling is applied, the model not only achieves higher overall predicted scores but also maintains stable performance across increasing guidance scales. This shows that the resampling strategy effectively improves generation quality by introducing multiple refinement opportunities. Moreover, the use of hybrid guidance resampling further enhances robustness and avoids overfitting to noisy classifier gradients and preserves the plausibility of the generated sequences. Consequently, resampling stabilizes performance across guidance scales, ensuring plausibility and functional feasibility of generated peptides.

*Contribution of individual classifier modules.* To assess the necessity and effectiveness of each constraint-specific classifier, we conduct ablation experiments by selectively disabling parts of the multi-objective classifier. The results are shown in Table 2, where we evaluate generation quality using plDDT${\;}^{p}$ and antigenicity score. In addition, we also evaluate the MHC binder percentage, which represents the proportion of generated peptides predicted as strong binders to the target MHC allele by NetMHCpan (Reynisson *et al.* 2020).

**Table 2:** Ablation study on the contribution of different classifier modules.

Module	plDDT${\;}^{p}$ ↑	Antigenicity score ↑	MHC binders (%)↑
unconditional	0.7182	0.2486	13.90
TCR-p only	0.7178	0.1784	9.01
pMHC only	0.7258	0.4537	31.89
Epitope only	0.7115	0.2096	12.46
TCR-p and pMHC	0.7243	0.3698	24.96
TCR-p and epitope	0.7084	0.1565	8.72
pMHC and epitope	0.7148	0.3804	26.97
All classifiers	**0.7443**	**0.4723**	**37.55**

The best result in each column is shown in bold and the second-best result is underlined. ↑ indicates that a higher value is better.

Among the individual modules, the pMHC classifier contributes most significantly to structural quality, which is biologically reasonable given that peptides must strictly conform to the MHC binding groove to ensure complex stability. This module also benefits from a substantially larger training dataset compared to other modules, enabling it to capture more accurate and generalizable constraints. Nevertheless, while the pMHC module alone achieves strong structural metrics, incorporating all three modules yields the best overall performance, reflecting the inherent cooperative nature of TCR-pMHC recognition. The TCR classifier ensures that peptides are compatible with TCR engagement, and the antigenicity module promotes sequences likely to trigger immune activation. This result highlights the complexity of the TCR-pMHC binding process, suggesting that no single constraint is sufficient to capture the full range of interactions. Instead, modeling multiple objectives simultaneously is essential for generating peptides that satisfy the structural and functional requirements of TCR-pMHC complex formation. We analyze the trajectory of gradient norms to demonstrate that all classifiers contribute dynamically without overweighting any single constraint. Results are shown in Supplementary Information S3.5, available as supplementary data at *Bioinformatics* online.

### 3.4 EPIC adapts peptide generation to context-specific immune environments

*Peptide naturalness.* To evaluate the naturalness of generated peptides, we compare the amino acid frequency distributions of EPIC-generated peptides with those observed in real antigenic epitopes. Figure 3D illustrates the amino acid composition from unconditional generation against the native distribution, along with their difference profiles, while Fig. 3E shows the corresponding comparison under condition-guided generation. The mean absolute error (MAE) between the generated and native distributions was remarkably low in both settings (unconditional: 0.0081; conditional: 0.0157), indicating that EPIC effectively captures the global amino acid usage patterns inherent to natural epitopes. Notably, even under conditional generation, where the binding constraints imposed by specific TCR-MHC contexts may inherently bias sequence composition, the distribution shift remains minimal. This consistency suggests that EPIC maintains strong biological plausibility while flexibly adapting to the biochemical requirements of specific immune recognition contexts.

*Context-aware adaptation.* To further examine whether EPIC adapts its generation process to context-specific binding environments, we conduct a systematic analysis across 83 distinct TCR-MHC test cases, each generating 1000 peptides. For each pair of test cases, we quantify the shift in peptide composition by computing mean position-wise Jensen-Shannon distance (${\bar{D}}_{JS}$) between their generated sequences. Let $P_{i}$ and $Q_{i}$ be the amino acid probability vectors at position *i* for two generated peptide sets of length *L*. The metric is defined as the average of the position-specific distances:


$${\bar{D}}_{JS}(P,Q)=\frac{1}{L}\sum_{i=1}^{L}\sqrt{\text{JSD}(P_{i}||Q_{i})},$$

where $\text{JSD}(P_{i}||Q_{i})=\frac{1}{2}D_{KL}(P_{i}||M_{i})+\frac{1}{2}D_{KL}(Q_{i}||M_{i})$ is the Jensen-Shannon Divergence at position *i*, and $M_{i}$ is the average distribution. Unlike KL divergence, ${\bar{D}}_{JS}$ constitutes a symmetric metric satisfying the triangle inequality, allowing for consistent geometric interpretation in correlation analyses. As shown in Fig. 3C, the resulting distributional distances exhibit clear variation across TCR-MHC contexts. Importantly, a significant negative correlation is observed between the JSD-based distances and TCR-MHC sequence similarity (Spearman’s ρ=−0.480,p<0.001), indicating that immune contexts with higher sequence similarity tend to yield more similar peptide composition patterns. This pattern aligns with the principle of context-dependent antigen recognition that TCRs sharing structural or sequence similarity often recognize peptides with conserved physicochemical motifs presented by related MHC alleles. Conversely, distinct TCR-MHC contexts favor unique sequence signatures, reflecting the immune system’s capacity to diversify recognition while preserving cross-reactivity within related contexts. Collectively, these results demonstrate that EPIC not only captures the global characteristics of natural peptide distributions but also dynamically adjusts its generative behavior in response to specific TCR-MHC contexts, reflecting strong context-aware adaptability in the design process. Hence, EPIC’s context-aware adaptability represents a key step toward biologically faithful modeling of immune specificity and cross-reactivity in peptide design. We perform a motif consistency analysis to further verify that generated peptides adhere to canonical binding rules and context-specific variations. Results are shown in Supplementary Information S3.6, available as supplementary data at *Bioinformatics* online.

## 4 Discussion

In this study, we present EPIC, a biologically interpretable and computationally efficient framework for *de novo* epitope generation. By decomposing the TCR-pMHC recognition process into antigenicity, MHC presentation, and TCR recognition, EPIC effectively integrates heterogeneous datasets and enables precise control over biological constraints. This biologically grounded formulation achieves a strong balance between novelty, structural plausibility, and functionality, surpassing end-to-end and LLM-based baselines across sequence, structure, and functional metrics.

Distinct from conventional paradigms that optimize isolated objectives, EPIC jointly models the interdependent processes governing antigen presentation and immune recognition. Its divide-and-conquer framework encodes each biological principle through modular classifiers while maintaining global coherence via guided diffusion. This design enhances both controllability and generalization. It also reveals interpretable biological behavior, such as context-dependent sequence adaptation that aligns with TCR-MHC compatibility. This highlights that EPIC captures genuine immunological mechanisms rather than superficial sequence patterns. Although LLMs also achieve reasonable performance in this task, their best results often coincide with or closely resemble known epitopes, revealing limited capacity to explore novel antigenic space. In contrast, EPIC generates highly diverse peptide candidates that maintain strong structural plausibility and immunological relevance.

Admittedly, the conditional independence assumption used to factorize the guidance objectives (Equation 6) is an approximation of the physical coupling within the TCR-pMHC complex. While this factorization is a methodological necessity to ensure computational tractability given the inherent sparsity of paired triplet data, it effectively models the primary constraints of the interface. Although specific edge cases involving complex steric clashes may exist, our empirical analysis demonstrates that this approximation is robust for the vast majority of designs. As detailed in Supplementary Information S4.1, available as supplementary data at *Bioinformatics* online, we observe a consistent positive correlation between classifier guidance scores and structural confidence metrics, confirming that satisfying these decomposed constraints serves as a reliable proxy for achieving overall physical compatibility.

Another limitation is data scarcity and potential sequence homology leakage during classifier evaluation. The paired single-cell datasets contain too few unique epitopes for strict cluster-based splitting, so stratified splits inevitably create partial overlap between test and training neighborhoods. Predictive metrics of individual classifiers, therefore, reflect generalization to unseen TCRs or near-duplicate peptides rather than strictly out-of-distribution sequences. Nevertheless, the generative model itself does not merely memorize training sequences: only 0.72% of EPIC-generated peptides share ≥80% sequence identity with the training set (Supplementary Information S4.2, available as supplementary data at *Bioinformatics* online), indicating that it learns underlying binding patterns.

Regarding experimental validation, we acknowledge that our assessment currently relies on established computational proxies, including AlphaFold3 confidence scores and Rosetta interface energies. While we recognize that *in silico* metrics cannot fully replace *in vivo* assays, these tools represent the current state-of-the-art for rigorous theoretical validation in peptide design. Given that large-scale wet-lab screening remains a significant bottleneck for the iterative evaluation of generative architectures, our study focuses on establishing EPIC-generated epitopes as high-fidelity candidates. By significantly narrowing the vast search space of the TCR-pMHC triplet, our framework provides a principled foundation for future high-throughput experimental verification, bridging the gap between generative modeling and clinical application.

EPIC provides new insights into the development of rapid and reliable neoantigen discovery for precision immunotherapy. In future work, we will integrate EPIC’s generative framework with *in vitro* cellular binding assays to identify peptides with high immunogenicity and functional relevance. Furthermore, as the protein design field increasingly shifts from sequence-based approaches toward structure-sequence co-design, the scarcity of experimentally validated TCR-pMHC complex structures remains a critical bottleneck. Expanding experimentally validated high-quality triplet datasets and structural benchmarks will be key to advancing accurate modeling of TCR-pMHC interactions and unlocking the full potential of precision immunotherapy design.

## Supplementary Material

btag358_Supplementary_Data
