# Supplementary material for: EPIC: multi-objective guided diffusion for epitope design in TCR-pMHC complexes
Source: Bioinformatics. 2026 Jun 4;42(6):btag358. doi: 10.1093/bioinformatics/btag358 (PMC13282085; doi:10.1093/bioinformatics/btag358)
Supplement: btag358_Supplementary_Data [file btag358_supplementary_data.pdf]

Supplementary Information

EPIC: Multi-objective Guided Diffusion for Epitope Design in TCR-pMHC Complexes

Yueshan Huang[a,1], Gufeng Yu[a,1], Letian Chen[a,b,1], Haoyang Luan[a], Yang Yang[a,*]

[a] *AGI Institute, School of Computer Science, Shanghai Jiao Tong University, 800 Dongchuan Road, Shanghai, 200240, China*

[b] *Shanghai Innovation Institute, No. 3, Lane 699, Huafa Rd., 200231, Shanghai, China*

1. These authors contributed equally to this work.

E-mail: yangyang@cs.sjtu.edu.cn

List of Contents

S1. Details of model 3

S1.1 Model architecture 3

S1.2 Training details 3

S1.3 Sampling details 4

S2. Details of experimental setups 4

S2.1 Datasets 4

S2.2 Baselines 6

S2.3 Metrics 8

S3. Details of experimental results 11

S3.1 Comparison results 11

S3.2 Case study 14

S3.3 Impact of generative backbone 16

S3.4 Comparison with latent diffusion on ESM embeddings 16

S3.5 Analysis of gradient balancing during generation 17

S3.6 Motif analysis 18

S4. Limitations 19

S4.1 Methodological limitations 19

S4.2 Data limitations and homology leakage 21

S1. Details of model

S1.1 Model architecture

Our generator adopts a 1D U-Net architecture built upon residual blocks for denoising one-hot encoded peptide sequences. The input combines peptide embeddings with sinusoidal time embeddings, and is projected into a hidden size of 768. The network consists of three downsampling residual blocks in the encoder, two residual blocks in the bottleneck, and three upsampling stages in the decoder. All convolutions use kernel size 3, with batch normalization and ReLU activation. The denoising model is integrated into a VP-SDE framework with $T = 1000$ and a linear noise schedule.

Discriminative classifiers for different biological sub-objectives share the same architecture. Each model is built on top of a pretrained ESM-2 encoder (`esm2_t6_8M_UR50D`)[1], which produces contextualized token representations for the input sequence(s). After obtaining the per-residue embeddings, we apply mean pooling over valid tokens to obtain fixed-length sequence-level embeddings. For multi-input cases (e.g., TCR and peptide), we concatenate the pooled embeddings before feeding them into a two-layer MLP classifier with hidden dimension 256 and ReLU activation.

S1.2 Training details

Training the diffusion-based generator. The generator is trained following the standard VP-SDE formulation[2] in the continuous relaxation of the one-hot encoded peptide space.

Since the generator is unconditional, its training can be conducted using a large corpus of known peptide sequences in IEDB[3], without requiring matching TCR or MHC information. This alleviates the data scarcity problem in TCR-pMHC triplets and enables stable pretraining of the generative backbone.

Training the discriminative classifiers. Each of the three classifiers is independently trained in a supervised manner using binary cross-entropy (BCE) loss. This design decouples the training of classifiers from the generator and allows the use of heterogeneous datasets with high-quality supervision. Each classifier focuses on a specific sub-task with well-defined biological interpretation, improving both training efficiency and interpretability.

Training configurations. We use the Adam optimizer with a fixed learning rate of 1×10^{-4}. For the diffusion generator, we follow the standard VP-SDE setup with $T = 1000$. During training, a timestep t is uniformly sampled from $[1, T]$ for each input, and noise is added accordingly. The noise schedule β_t is defined as a linear interpolation between 10^{-4} and 0.02.

S1.3 Sampling details

Resampling process. During the last 400 timesteps ($t \leq 400$), every 10 diffusion steps we perform $K = 10$ rounds of resampling. The resampling procedure operates as follows:

1. **Noise injection:** We perturb the current peptide state by adding noise, effectively reverting it to its state 10 steps earlier in the diffusion chain.
2. **Denoising:** For $k = 1, \ldots, K - 1$, we apply gradient-guided denoising. For $k = K$, we perform an unconditional denoising step without classifier gradients, relying solely on the learned generative prior.

Sampling configurations. We perform generation with $T = 1000$ diffusion steps. For timesteps $t \geq 400$, the model performs unconditional denoising using only the pretrained generator. For $t < 400$, classifier guidance is applied to steer the generation toward satisfying biological constraints with guidance scale $\eta = 50.0$.

S2. Details of experimental setups

S2.1 Datasets

Following previous studies[4,5], we use three curated datasets to support the training of individual components in EPIC and the evaluation of its overall performance. A summary of the datasets is provided in Table S1.

Table S1. Summary of datasets used for training and evaluation

Dataset	Source	Positive	Negative	Total
TCR-pMHC triplet dataset	10x Genomics[6]	953	15,793	16,746
Peptides dataset	IEDB[3]	8,793	17,401	26,194
pMHC binding dataset	BigMHC[7]	333,437	17,639,877	17,973,314

TCR-pMHC triplet dataset. We collect TCR-pMHC triplet data from the BEAM-T dataset released by 10x Genomics[6]. BEAM-T (Barcode-enabled Antigen Mapping for T cells) is a large-scale, single-cell immune profiling dataset that provides paired full-length TCR$\alpha\beta$ sequences along with MHC allele types, epitopes, and associated TCR-pMHC specificity scores derived from antigen barcoding. Specifically, we aggregate data from five BEAM-T datasets:

- 5k Human A0201 | B0702 PBMCs
- 2k Mouse H2Kb OT-1 Splenocytes
- 10k Human A1101 PBMCs with EBV spike-in
- 10k Human A2402 PBMCs with EBV and CMV spike-in
- 10k Human A0201 PBMCs with CMV, Flu, and SARS-CoV-2 spike-in

TCR α and β chain sequences from the same cell are matched using the barcode field. Only TCRs for which both chains are present and have complete variable (V), diversity (D), joining (J) and constant (C) regions are retained, with all samples containing missing or incomplete chains being discarded.

We use the Cell Ranger software[8] provided by 10x Genomics to compute the antigen specificity score for each cell, which quantitatively reflects the likelihood of specific TCR-antigen binding. This score is derived primarily from the antigen UMI count and normalized by comparing the signal intensity with background control probes and the relative abundance of different antigen probes within the same cell The resulting score ranges from 0 to 1. To obtain labels for training the TCR-p specificity classifier, we binarize the specificity scores using a threshold of 0.5.

Therefore, each data entry contains four components: a full-length TCR sequence (paired α and β chains), an MHC allele, an epitope, and a specificity label. After deduplication, the final dataset contains 953 positive and 15,793 negative TCR-pMHC triplets, covering 7,947 unique full-length TCRs and 10 epitopes.

Peptides dataset. To train the antigenicity classifier and pretrain the unconditional generator, we collect peptide data from the Immune Epitope Database (IEDB)[3]. We filtered the linear peptide sequences within the length of 8-13 amino acids of T cell and MHC Class I assays and Host: Homo sapiens (human). We remove duplicate sequences and standardize the amino acid vocabulary.

Based on the qualitative measurement annotations (Positive, Positive-High, Positive-Intermediate, Positive-Low, Negative), peptides were categorized into positive and negative samples. Since certain peptides may elicit immune responses when presented by specific MHC alleles but not by others, we excluded such ambiguous cases to remove inconsistency. Specifically, only peptides that consistently induced immune responses across all recorded MHC contexts were retained as positive samples, while those that consistently failed to elicit responses under all tested MHCs were designated as negative samples. The final dataset includes 8,793 positive and 17,401 negative peptides.

pMHC binding dataset. The training and validation data for predicting epitope presentation is provided by BigMHC[7], which consisted of 333,437 positive peptide-MHC pairs and 17,639,877 negative pairs, across 149 MHC alleles.

Data splitting. All these datasets exhibit significant class imbalance. To ensure fair training and evaluation, we apply stratified sampling to split each dataset into training and test sets at a 9:1 ratio. In addition, since the TCR-pMHC triplet dataset also shows substantial imbalance across different peptide classes, we further stratify the split based on peptide identity to preserve epitope-level distribution. During generation, only positive samples are used for training the unconditional diffusion model, while both positive and negative data contribute to classifier learning.

S2.2 Baselines

For peptide generation based on specialized TCR and MHC, we compare our method against several baseline methods, including LLMs and specialized models. The LLMs include **ChatGPT-4o**[9], **DeepSeek-v3**[10], and **Gemini-2.5-flash**[11]. These models are advanced LLMs that have demonstrated strong generative capabilities in scientific domains, including biology and medicine. To maximize their performance, we carefully designed prompts and conducted both zero-shot and few-shot learning. The prompt details are shown in Table S2.

EpiGen[12] is originally designed for TCR-specific epitope generation, where the complementarity-determining region 3 of the TCR β chain (CDR3β) serves as the sole input feature. Following the original implementation, we use the TCR CDR3β sequences extracted from each TCR-pMHC triplet as input, and the model outputs the corresponding peptide candidates. Since EpiGen does not incorporate MHC information, its generated peptides are independent of MHC context.

PepPPO[13] is a reinforcement learning-based framework for optimizing peptides that bind specific MHC alleles. We employ the model in its MHC-conditioned generation mode, where the input is the target MHC allele sequence. The sampling rate is set to 0.5 following the original paper, and the output peptides are optimized toward the binding affinity objective defined in PepPPO.

PepTune[14] is a multi-objective discrete diffusion model that utilizes Monte Carlo Tree Guidance to optimize peptide sequences. In our implementation, we train the underlying masked discrete language model on our curated peptide dataset to capture the general distribution of epitope sequences. During the inference phase, we employ the predicted probabilities from our pre-trained antigenicity, MHC presentation, and TCR specificity classifiers as the reward signals. The guidance algorithm uses these rewards to balance exploration and exploitation, iteratively refining the generated sequences toward the desired biological objectives.

Table S2. Prompts for generating peptides used in LLMs

Learning method	Prompt
Zero-shot	You are a protein design expert specialized in T-cell receptor (TCR) and peptide-MHC (pMHC) complex modeling. I will provide a full-length paired TCR sequences (alpha chain and beta chain) and a full-length MHC protein sequence. Your task is to design a peptide (8-15 amino acid length) that can be presented by the given MHC molecule and recognized by the provided TCR. Only output the peptide amino acid sequence in uppercase on a single line. Do not include any explanation or additional text.<br>• TCR alpha chain: {}<br>• TCR beta chain: {}<br>• MHC sequence: {}
Few-shot	You are a protein design expert specialized in T-cell receptor (TCR) and peptide-MHC (pMHC) complex modeling. I will provide a full-length paired TCR sequences (alpha chain and beta chain) and a full-length MHC protein sequence. Your task is to design a peptide (8-15 amino acid length) that can be presented by the given MHC molecule and recognized by the provided TCR. Here are two examples:<br>Example 1:<br>TCR alpha chain: MTRVSL......VYQLRD<br>TCR beta chain: MGPGLL......AVFEPS<br>MHC sequence: MLVMAP......DVSLTA<br>Generated peptide: TPRVTGGGAM<br>Example 2:<br>TCR alpha chain: MEKNPL......VYQLRD<br>TCR beta chain: MSIGLL......AVFEPS<br>MHC sequence: MAVMAP......LTACKV<br>Generated peptide: GILGFVFTL<br>Only output the peptide amino acid sequence in uppercase on a single line. Do not include any explanation or additional text.<br>• TCR alpha chain: {}<br>• TCR beta chain: {}<br>• MHC sequence: {}

MOG-DFM[15] presents a framework for steering discrete-time flow matching generators toward Pareto-efficient tradeoffs across multiple scalar objectives. Similar to the setup for PepTune, we first train the unconditional discrete flow matching base model on our peptide dataset. We then apply the adaptive hypercone filter mechanism during the sampling process. The outputs from our three classifiers serve as the scalar objectives, enabling the model to filter candidate transitions and steer the generation trajectory toward sequences that simultaneously satisfy the distinct immunological constraints.

PepMLM[16] is a general masked language modeling framework for designing peptide binders against target proteins. In our experiments, we employ the `esm2-t33-650M-UR50D` backbone as the base model and fine-tune it on our curated TCR-pMHC triplet dataset to adapt it for immunological epitope design. For input representation, we concatenate the full-length sequences of both TCR chains and the corresponding MHC molecule, using the ESM separator token (".") to delimit each segment. The model is trained to autoregressively generate peptide sequences conditioned on the combined TCR-MHC context.

DPLM[17] is a protein design model that supports sequence-conditioned generation. We use the `dplm-150m` variant in its sequence-conditioned (inpainting) mode, where the model fills in missing regions (masked residues) given contextual sequences. Specifically, we concatenate the TCR, peptide, and MHC sequences separated by the "." token. The TCR and MHC regions are designated as conditioning positions, while the peptide region is masked for inpainting. This setup enables DPLM to generate epitope sequences consistent with the TCR-MHC binding context.

S2.3 Metrics

Several metrics are employed to comprehensively evaluate the performance of epitope generation. **Sequence-level metrics** assess the model's ability to produce novel and diverse peptides, while **structure-level metrics** evaluate the plausibility of the generated peptides to form stable TCR-pMHC complexes. In addition, **functional metrics** are used to estimate the likelihood that generated peptides can be effectively presented by MHC molecules and recognized as immunogenic binders. As existing predictors still lack sufficient accuracy for modeling full TCR-pMHC binding affinity, structure-level and pMHC-level functional metrics are used as reasonable alternatives to evaluate epitope antigenicity and binding potential. The details of these evaluation metrics are described below.

For sequence-level evaluation:

- **Similarity** score assesses the alignment score between generated peptide sequences and the ground truth sequence within the corresponding TCR-pMHC triplet. A lower score indicates greater novelty in the generated sequences. This score is calculated using the PairwiseAligner from the Biopython package[18]. Additionally, the **Novelty** score reflects the degree of dissimilarity in the alignment, with higher scores indicating more novel sequences.
- **Diversity** score reflects the dispersion of generated sequences by pairwise alignment scores. A higher score indicates the ability to generate many distinct sequences for a given TCR and MHC. This score is also calculated using the PairwiseAligner from the Biopython package.
- **Uniqueness** score measures the proportion of unique sequences among the generated peptides. A higher score means more diverse sequences are generated for a given TCR and MHC. This score is computed directly by calculating the percentage of unique sequences relative to the total number of generated sequences.

For structure-level evaluation:

- **plDDT (Predicted Local Distance Difference Test)** score indicates the model's confidence in the accuracy of the predicted structure for each residue. Higher plDDT scores suggest higher confidence in the predicted structure.
- **plDDT^{p}** refers to the plDDT score specifically for the peptide segment in the predicted structure.
- **ipTM (Interface Predicted Template Modeling)** score evaluates the quality of the predicted interface between two interacting proteins. It measures how well the predicted interface aligns with known interfaces in the template structures.
- **pTM (Predicted Template Modeling)** score assesses the overall quality of the predicted structure by comparing it to known template structures. It provides a measure of how well the predicted structure matches the templates used for modeling.
- **actifpTM (Actual Interface pTM)** score[19] evaluates the predicted active interface regions in protein-protein interactions. It helps in identifying the key residues involved in the interaction and their predicted accuracy.

During testing, we use tFold-TCR[20] to predict the TCR-pMHC structures and calculate all the above structure-level metrics.

For functional evaluation:

- **Affinity score** quantifies the predicted binding strength between a peptide and a given MHC allele, as estimated by the MHCflurry binding affinity (BA) predictor[21]. Lower values correspond to tighter peptide-MHC binding, indicating higher binding affinity and stronger potential for stable complex formation.
- **Affinity percentile** represents the normalized binding affinity rank of a peptide among random peptides for the same MHC allele. Lower percentile values indicate stronger predicted binders. This metric allows for comparison of binding strengths across different alleles with varying baseline affinities.
- **Processing score** evaluates the likelihood that a peptide can be correctly processed and transported through the antigen presentation pathway, as predicted by the MHCflurry antigen processing (AP) model[21]. Higher values suggest that the peptide sequence is more compatible with proteasomal cleavage, TAP transport, and ER trimming signals.
- **Presentation score** provides an integrated prediction of peptide presentability on the cell surface, combining binding affinity (BA) and antigen processing (AP) predictions through a logistic regression model[21]. Higher presentation scores indicate a higher probability that the peptide will be successfully processed, bound, and displayed by MHC molecules. We consider this metric to be highly representative of the peptide's overall antigenic potential, and therefore refer to it as the **antigenicity score** in Table 1.
- **Presentation percentile** denotes the rank-normalized version of the presentation score across a background peptide distribution. Lower values indicate higher predicted presentability, facilitating cross-allele comparisons of peptide-MHC presentation likelihood.
- **EL score** represents the predicted likelihood that a peptide can be presented as an MHC ligand, as estimated by NetMHCpan[22]. This score integrates both peptide-MHC binding affinity and antigen processing information. A higher EL score indicates a greater probability that the peptide will be naturally presented on the cell surface.
- **EL score rank** denotes the percentile rank of the EL score among all peptides for the same MHC allele A lower rank value corresponds to a higher presentation likelihood, providing a normalized measure that facilitates cross-allele comparison.
- **BA score** reflects the predicted binding affinity between a peptide and an MHC molecule, computed by NetMHCpan based on experimental binding affinity data. Higher BA scores correspond to tighter peptide-MHC

interactions, which are essential for stable complex formation and effective presentation.

- **BA score rank** represents the percentile rank of the BA score, relative to a reference peptide distribution for the same MHC allele. Lower BA ranks indicate stronger predicted binding and higher binding confidence.
- **Strong binders percentage** measures the proportion of peptides predicted by NetMHCpan as strong MHC binders, typically defined as those with a BA rank below 0.5%. A higher percentage indicates that a greater fraction of generated peptides are expected to form stable peptide-MHC complexes.
- **Weak binders percentage** quantifies the proportion of peptides predicted by NetMHCpan as weak binders, typically defined by a BA rank between 0.5% and 2.0%. This metric complements the strong binders percentage, reflecting the overall binding distribution across generated peptides.

S3. Details of experimental results

S3.1 Comparison results

Table S3 reports all structure-level metrics for EPIC and the baselines. Compared to the baselines, our method achieves significant improvements in three metrics ($plDDT^p$, ipTM, and actifpTM) related to the stability and accuracy of the TCR-pMHC complex interface.

Table S3. Performance comparison between EPIC and baseline methods on structure-level metrics

Methods	plDDT^p ↑	ipTM ↑	pTM ↑	actifpTM ↑
Random sampling	0.6837	0.8044	0.8335	0.7411
ChatGPT 4o (zero-shot)	0.6527	0.8037	0.8330	0.7414
ChatGPT 4o (few-shot)	0.7256	0.8048	0.8335	0.7409
DeepSeek-v3 (zero-shot)	0.6322	0.8038	0.8331	0.7403
DeepSeek-v3 (few-shot)	0.7160	0.8047	**0.8361**	0.7411
Gemini-2.5-flash (zero-shot)	0.7303	0.8039	0.8329	0.7387
Gemini-2.5-flash (few-shot)	0.7343	0.8046	0.8336	0.7383
EpiGen	0.6483	**0.8049**	0.8357	0.7415
PepPPO	0.7008	0.8041	0.8331	0.7410
PepTune	0.6989	0.8021	0.8316	0.7397
MOG-DFM	0.6869	0.8005	0.8307	0.7398
PepMLM	0.7276	0.8026	0.8318	0.7338
DPLM	0.7047	0.8035	0.8326	0.7398
EPIC (unconditional)	0.7182	0.8047	0.8336	0.7409
EPIC (conditional)	**0.7443**	**0.8049**	0.8336	**0.7418**

Table S4 and Table S5 summarize the results for EPIC and all baseline methods across functional metrics related to peptide and pMHC properties. Overall, EPIC achieves consistently strong performance across all evaluation metrics, ranking second only to PepPPO, which explicitly optimizes peptide sequences using these metrics as reinforcement learning rewards. The peptides designed by EPIC exhibit high predicted affinity toward their corresponding MHC molecules and demonstrate strong binding potential, characterized by higher proportions of both strong binders and weak binders compared with other generative models.

Table S4. Performance comparison between EPIC and baseline methods on MHCflurry2.0 metrics

Methods	Affinity ↓	Affinity percentile ↓	Processing score ↑	Presentation score ↑	Presentation percentile ↓
Random sampling	20226.15	20.60	0.1441	0.0628	45.09
ChatGPT 4o (zero-shot)	14836.49	14.28	0.3347	0.1971	19.40
ChatGPT 4o (few-shot)	10067.21	7.20	0.3055	0.3257	15.57
DeepSeek-v3 (zero-shot)	16051.37	14.81	0.2403	0.1435	28.34
DeepSeek-v3 (few-shot)	13894.12	10.05	0.3552	0.1987	16.57
Gemini-2.5-flash (zero-shot)	8858.21	6.40	0.2920	0.3847	12.93
Gemini-2.5-flash (few-shot)	7516.29	5.28	0.2477	0.4301	12.23
EpiGen	22029.36	23.53	0.1741	0.0617	45.35
PepPPO	**259.65**	**0.49**	**0.5379**	**0.8628**	**0.25**
PepTune	23078.62	26.97	0.0612	0.0283	62.02
MOG-DFM	29506.97	59.24	0.2612	0.0212	46.79
PepMLM	20503.44	15.77	0.0503	0.0177	58.89
DPLM	20363.88	18.30	0.0771	0.0409	54.03
EPIC (unconditional)	10851.83	8.18	0.2859	0.2486	16.58
EPIC (conditional)	<u>5150.68</u>	<u>3.59</u>	<u>0.3533</u>	<u>0.4723</u>	<u>6.91</u>

Table S5. Performance comparison between EPIC and baseline methods on NetMHCpan metrics

Methods	EL score ↑	EL score rank ↓	BA score ↑	BA score rank ↓	Strong binders percentage ↑	Weak binders percentage ↑
Random sampling	0.0248	35.02	0.1241	35.23	2.04	4.03
ChatGPT 4o (zero-shot)	0.1071	16.09	0.2276	19.22	10.84	10.38
ChatGPT 4o (few-shot)	0.2012	12.60	0.3129	15.03	21.56	13.35
DeepSeek-v3 (zero-shot)	0.0727	22.37	0.1778	25.35	7.41	8.63
DeepSeek-v3 (few-shot)	0.1142	17.93	0.1906	24.44	12.36	5.89
Gemini-2.5-flash (zero-shot)	0.2649	10.43	0.3367	15.43	29.64	16.33
Gemini-2.5-flash (few-shot)	0.2920	8.57	0.3837	11.56	32.61	<u>19.80</u>
EpiGen	0.0289	32.11	0.1303	31.10	2.35	4.91
PepPPO	**0.5762**	**0.61**	**0.5981**	**1.56**	**68.91**	**25.12**
PepTune	0.0058	54.37	0.0876	41.39	0.25	1.50
MOG-DFM	0.0165	43.72	0.1841	28.98	0.49	5.20
PepMLM	0.0043	37.80	0.0372	60.70	0.19	0.54
DPLM	0.0169	38.16	0.0757	49.51	1.31	2.83
EPIC (unconditional)	0.1375	13.86	0.2634	17.66	13.90	13.04
EPIC (conditional)	<u>0.3418</u>	<u>5.77</u>	<u>0.4092</u>	<u>9.47</u>	<u>37.55</u>	17.74

S3.2 Case study

We present additional TCR-pMHC complex structures predicted by AlphaFold 3 for peptides designed by EPIC in Figure S1, further confirming the structural plausibility of our design and binding compatibility with the target TCRs and MHCs.

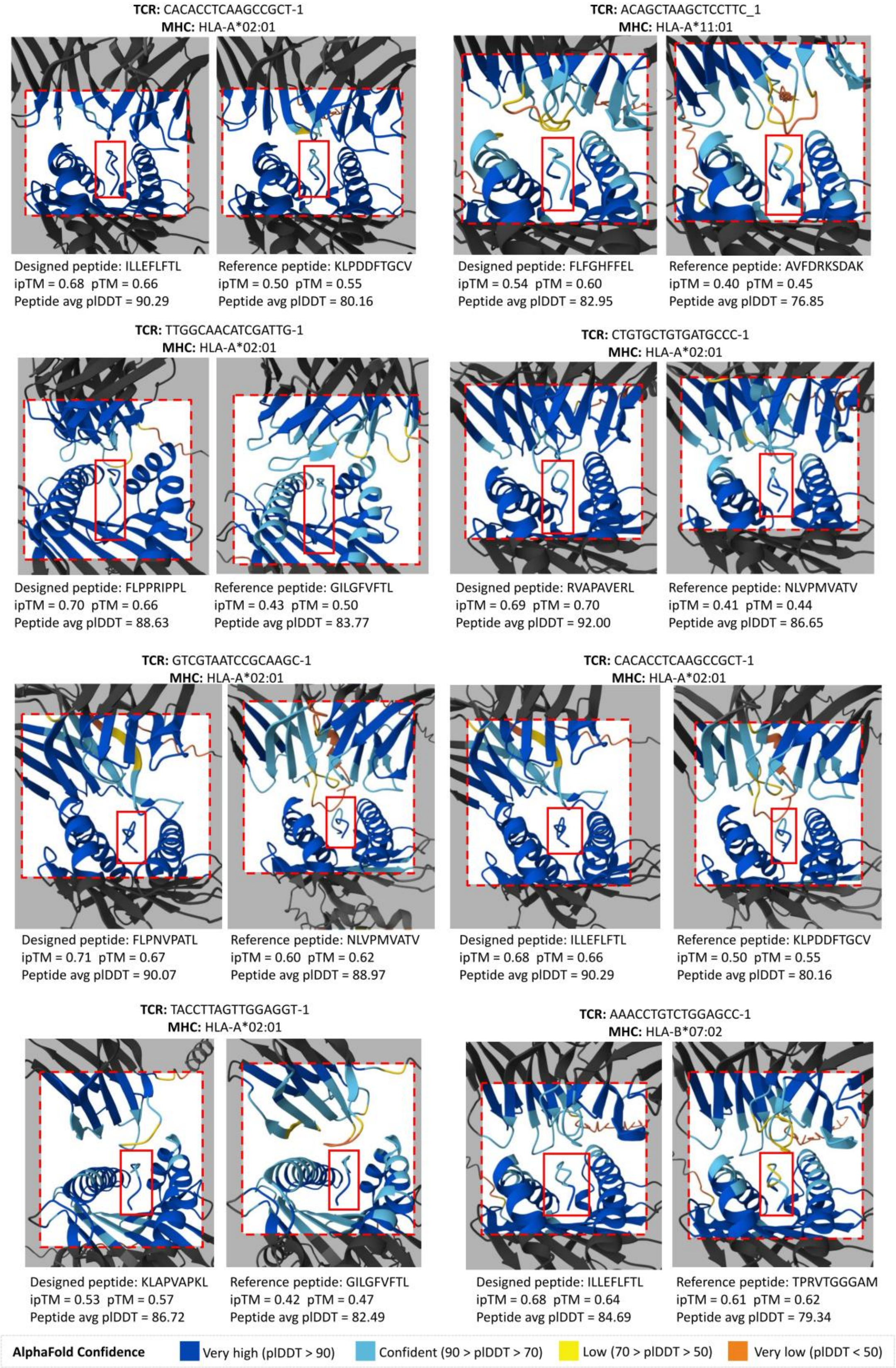

Figure S1. AlphaFold 3 structural prediction comparison between designed peptides and reference epitopes

S3.3 Impact of generative backbone

To investigate the influence of the generative modeling framework on epitope design performance, we conduct an ablation study comparing EPIC's continuous-time score-based diffusion with two discrete generative models: Discrete Denoising Diffusion Probabilistic Models (D3PM) and Discrete Flow Matching (DFM). Both discrete baselines are trained on the same peptide dataset as EPIC and utilize the same classifier-based guidance mechanism.

As shown in Table S6, while discrete models exhibit higher diversity in the unconditional setting, their performance improves negligibly or degrades when applying the multi-objective guidance constraints. Specifically, in the conditional setting, both discrete diffusion and DFM fail to achieve high antigenicity scores (0.0191 and 0.0144, respectively) or improve structural confidence (plDDT) compared to EPIC (0.4723 antigenicity, 0.7443 plDDT). This disparity suggests that the continuous relaxation employed in EPIC is critical for effectively integrating the fine-grained gradient signals backpropagated from the pre-trained classifiers. The discrete transition nature of the baselines limits the effectiveness of gradient-based guidance in this framework, resulting in generated sequences that do not satisfy the complex biological constraints of the TCR-pMHC triplet.

Table S6. Ablation study evaluating the impact of different generative backbones

Methods	Similarity ↓	Diversity ↑	Uniqueness ↑	$plDDT^p$ ↑	Antigenicity score ↑
EPIC (unconditional)	28.13	71.88	**100.00**	<u>0.7182</u>	<u>0.2486</u>
EPIC (conditional)	28.27	64.05	**100.00**	**0.7443**	**0.4723**
D3PM (unconditional)	22.57	77.55	**100.00**	0.6905	0.0197
D3PM (conditional)	22.19	77.58	**100.00**	0.6908	0.0191
DFM (unconditional)	<u>18.06</u>	<u>82.06</u>	**100.00**	0.6468	0.0131
DFM (conditional)	**15.89**	**84.91**	**100.00**	0.6473	0.0144

S3.4 Comparison with latent diffusion on ESM embeddings

To verify whether performing diffusion in a pre-trained protein language model latent space offers advantages over our residue-space approach, we implement a Latent Diffusion Model (LDM) baseline. We utilize the `esm2_t6_8M_UR50D` encoder to map peptide sequences into a continuous latent representation and use the pre-trained language modeling head as the decoder. A Transformer-based architecture is employed

as the denoising network, replacing the 1D U-Net used in EPIC, to better accommodate the latent features.

The performance comparison is presented in Table S7. While the latent diffusion model demonstrates reasonable performance in unconditional generation, it fails to respond effectively to the multi-objective guidance signals. This indicates that compressing sequences into a latent space may dilute the fine-grained, residue-specific information required for the classifiers to provide effective gradient guidance. By operating directly in the one-hot residue space, EPIC maintains precise control over sequence composition, allowing for more accurate optimization of the complex constraints inherent to TCR-pMHC interactions.

Table S7. Comparison between EPIC and a latent diffusion model operating on ESM embeddings

Methods	Similarity ↓	Diversity ↑	Uniqueness ↑	plDDT^p ↑	Antigenicity score ↑
EPIC (unconditional)	28.13	<u>71.88</u>	**100.00**	0.7182	<u>0.2486</u>
EPIC (conditional)	28.27	64.05	**100.00**	**0.7443**	**0.4723**
ESM LDM (unconditional)	<u>21.49</u>	**74.79**	99.84	0.7169	0.0526
ESM LDM (conditional)	**20.82**	71.04	98.76	<u>0.7189</u>	0.0629

S3.5 Analysis of gradient balancing during generation

To investigate whether the summation of gradients from different classifiers leads to the dominance of a single objective (overweighting) or effectively balances the multiple constraints, we track the L_2 norm of the gradients provided by the Antigenicity (f_1), MHC Presentation (f_2), and TCR Specificity (f_3) classifiers at each step of the reverse diffusion process.

Figure S2 illustrates the evolution of these averaged gradient magnitudes over the sampling trajectory from noise ($t = T$) to the final sequence ($t = 0$). We observe two key phenomena that support the stability of the multi-objective guidance:

1. Comparative Magnitudes: The average gradient norms for all three objectives remain within the same order of magnitude throughout the process. This confirms that the guidance signal is a balanced composite, and the optimization is not driven solely by the classifier with the sharpest local gradients.

2. Temporal Decoupling: The trajectories exhibit a coherent biological logic. The pMHC presentation gradient typically decreases as t approaches 0, implying that the hard constraints for MHC binding (e.g., anchor residues) are satisfied relatively early. In contrast, the antigenicity gradient often remains high in the final steps. This suggests a “structure-first, function-later” generation hierarchy: the model first resolves the structural compatibility with the MHC groove and then fine-tunes the surface residues to enhance antigenic potential.

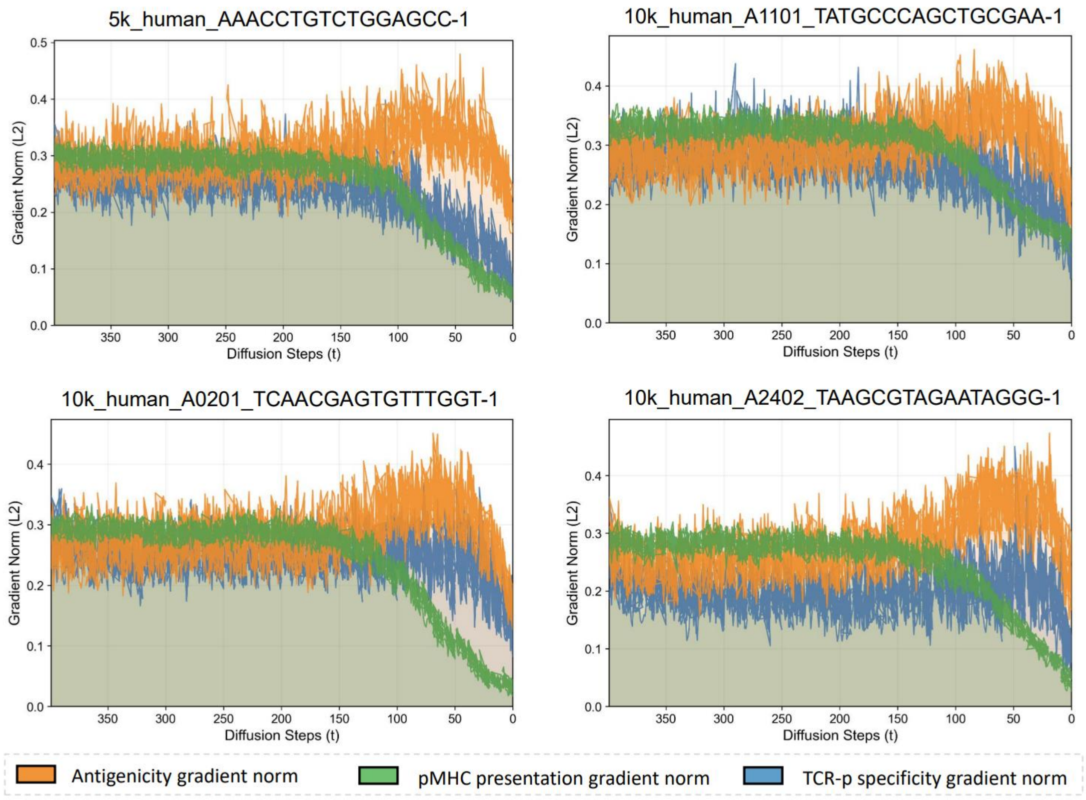

Figure S2. Trajectory of gradient norms during the reverse diffusion process

S3.6 Motif analysis

To verify that the high scores obtained by EPIC-generated peptides reflect genuine biological patterns rather than adversarial exploitation of the classifiers, we perform a motif analysis on a subset of 59 test cases (5,900 generated peptides) associated with the HLA-A*02:01 allele.

Figure S3 presents the sequence logos. As shown in Figure S3A, the global motif demonstrates strict compliance with the physical constraints of the MHC binding groove, showing the canonical preference for Leucine (L) at position 2 and Valine (V) or Leucine (L) at position 9.

Furthermore, to ensure the model adapts to specific TCR contexts, we compared the motifs of peptides generated for different TCRs targeting the same MHC allele. Figure S3B illustrates two representative cases. While both retain the required MHC anchors, their central regions (positions 4-8) differ significantly. The first case shows an enrichment of charged and polar residues (e.g., D, E, R), likely interacting with a complementary charged surface on the TCR. In contrast, the second case is characterized by bulky hydrophobic residues (e.g., L, F, W), suggesting a hydrophobic interaction interface. This variability confirms that EPIC generates epitopes that are not only MHC-compatible but also specifically tailored to the physicochemical properties of the target TCR.

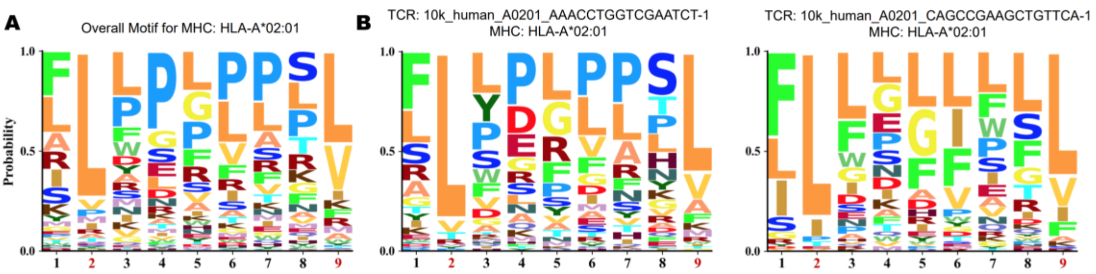

Figure S3. Motif consistency analysis for peptides generated under the HLA-A*02:01 allele constraint

S4. Limitations

S4.1 Methodological limitations

The guidance mechanism in EPIC factorizes the joint probability of TCR-pMHC binding into independent terms for antigenicity, MHC presentation, and TCR recognition (Eq. 6). As discussed in the main text, this independence assumption is a necessary computational strategy to leverage large-scale heterogeneous datasets and bypass the severe scarcity of paired triplet data. However, biologically, TCR recognition is physically coupled to MHC presentation, as the MHC molecule restricts the conformational space of the peptide. This simplification implies that c_T (TCR specificity) does not depend on m (MHC) once y (peptide) and r (TCR) are given, potentially ignoring steric clashes that arise only in the full triplet context.

To analyze the behavior when high classifier scores do not translate to physical stability, we examine a specific failure case from our top-3 generated candidates. As illustrated in Figure S4, the peptide `NLLGILNV` receives high scores from all three classifiers due to the presence of correct anchor motifs (L at pos 2, V at pos 8), yet AlphaFold3 predicts a low confidence score (plDDT^{p}=64.5). Structural inspection suggests that despite satisfying sequence motifs, the central residues may create steric clashes or backbone strain within the specific TCR-pMHC interface that are invisible

to 1D sequence models. We attribute this discrepancy to two factors: the inherent limitation of the independence assumption, and the imperfection of the classifiers themselves. Given the scarcity and class imbalance of the available training data, the decision boundaries of our predictors are not infallible. Consequently, the classifiers may occasionally yield false positives, assigning high confidence to sequences that satisfy learned sequence motifs but lack genuine binding capability. This underscores why we employ AlphaFold3 and Rosetta as essential downstream validation steps to filter out such outliers, and it motivates our future work to incorporate explicit structure-guided diffusion to resolve these issues.

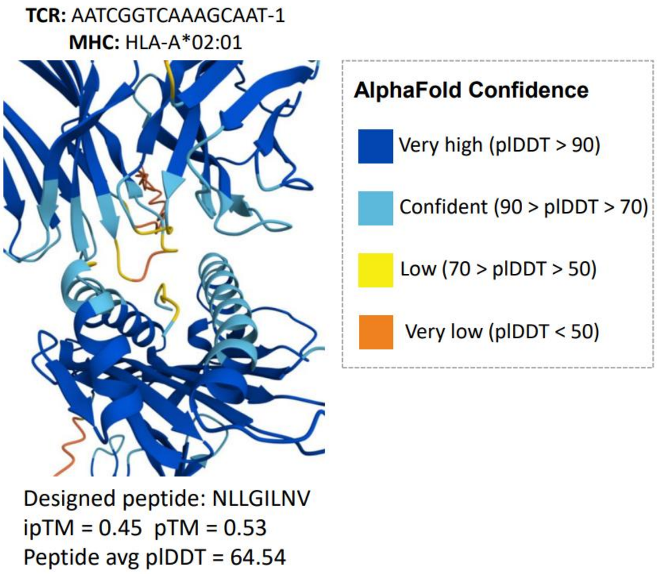

Figure S4. A representative case of sequence-structure discrepancy

We acknowledge this as a known limitation of sequence-level modeling. While the sequence-based factorization serves as a highly effective approximation for the vast majority of cases (as evidenced by the overall high success rates and Rosetta energies), it cannot fully account for 3D steric constraints. This highlights the importance of our downstream validation pipeline using AlphaFold and Rosetta, and it motivates our future work to incorporate explicit structure-guided diffusion to resolve these specific incompatibilities.

Despite these occasional outliers, we assess whether the independence approximation remains reasonable for the majority of cases. We analyze the correlation between our classifier guidance scores and the structural confidence metric (AlphaFold3 $plDDT^p$) across the test set. As shown in Figure S5, we observe a consistent positive correlation between the total score and $plDDT^p$. Notably, this correlation is largely driven by the pMHC presentation score (Pearson $r = 0.54$, Spearman $r = 0.58$). This alignment is biologically consistent, as the stability of the peptide-MHC complex serves as the structural prerequisite for the entire triplet; if the peptide does not fit the MHC groove, the complex cannot form stably. Furthermore,

results in Sec.III-B show that our top-ranked peptides achieve high average structural confidence and low Rosetta interface energies, which confirms that for the vast majority of generated sequences, satisfying the independent sequence constraints effectively translates to physical structural compatibility. These results show that this independence approximation is reasonable and effective in the majority of cases.

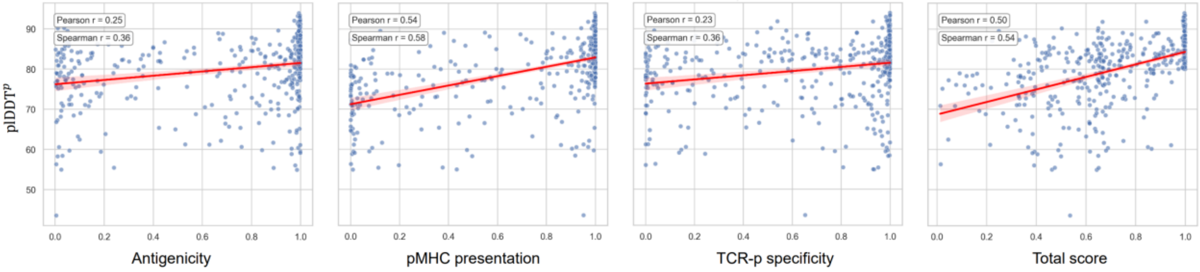

Figure S5. Scatter plots showing the correlation between classifier scores (Antigenicity, pMHC presentation, TCR-p specificity, and Total Score) and AlphaFold3 $plDDT^p$

S4.2 Data limitations and homology leakage

For sequence-based generative and predictive models, standard random or stratified data splits can sometimes lead to overly optimistic evaluations due to sequence homology leakage. To rigorously evaluate the boundaries of our framework and clarify the extent of data leakage versus generative extrapolation, we conducted a comprehensive homology-aware analysis. Sequence identity was calculated using global alignment without gap penalties (globalxx from Biopython, defined as matching residues divided by the maximum sequence length). Because standard protein homology thresholds (e.g., 30%–40%) are biologically uninformative for short 8–15mer peptides due to the mandatory presence of conserved MHC anchor residues, we defined high homology using a strict threshold of 80% sequence identity. This threshold strictly corresponds to allowing at most 1 to 2 amino acid mismatches.

We first acknowledge the potential data leakage in our discriminative classifiers, as strict cluster-based data splitting (e.g., ensuring no test peptide exceeds 80% identity to any training peptide) is highly challenging due to extreme data scarcity and the paired nature of the tasks. For the pairing tasks, including TCR specificity and pMHC presentation, the data splits were inherently limited by the available sequence diversity. The high-quality single-cell BEAM-T dataset contains over 16,000 paired data points but encompasses only 10 unique epitopes, making a peptide-level cluster-based split mathematically impossible. Similarly, the pMHC presentation dataset was split at the sequence-pair level, meaning a single peptide might appear in both the training and validation sets bound to different MHC alleles. Our overlap analysis revealed that 47.75%

of the unique positive peptides in the pMHC validation set also appear in the training set. For these modules, the stratified split evaluates the classifier's ability to learn interaction rules between paired molecules rather than purely out-of-distribution peptide generalization. For the peptide-level antigenicity task, we calculated the nearest neighbor sequence identity of each positive test sample against the positive training set. As shown in Figure S6, 36.48% of the test peptides share ⩾80% sequence identity with the training set. This indicates that the predictive test metrics for this module do partially benefit from localized sequence homology.

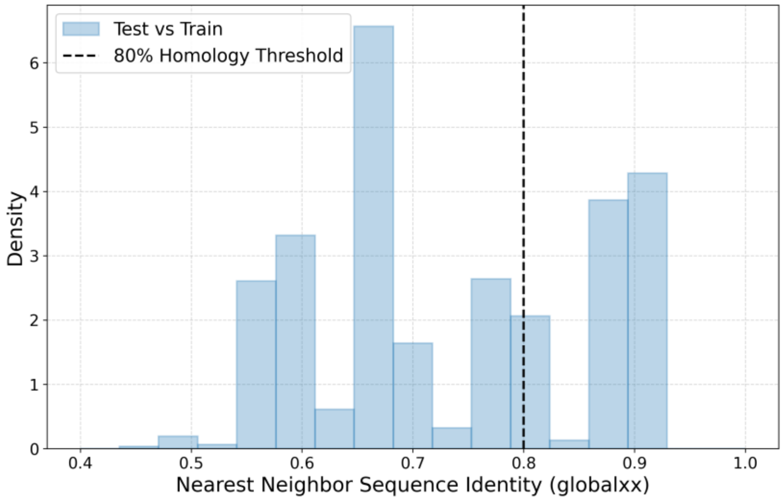

Figure S6. Distribution of Nearest Neighbor Sequence Identity for positive test peptides against the positive training set in the antigenicity dataset. The y-axis shows probability density normalized to unit total area, so the absolute values are arbitrary and meaningful only in shape comparison.

Although the individual classifiers are constrained by the localized homology of the training data, our analysis demonstrates that the final multi-objective guided diffusion process escapes these limits and does not merely memorize the training distributions. To objectively assess generative novelty, we established a biological baseline by computing the internal nearest neighbor identity within the training set itself (Intra-training-set identity). As shown in Figure S7, 39.02% of the training peptides share ⩾80% identity with other training peptides, reflecting the natural sequence redundancy driven by conserved MHC anchor residues. In contrast, only 0.72% of EPIC-generated peptides share ⩾80% identity with the training set. This near-zero leakage confirms that the generator effectively extrapolates beyond exact training examples.

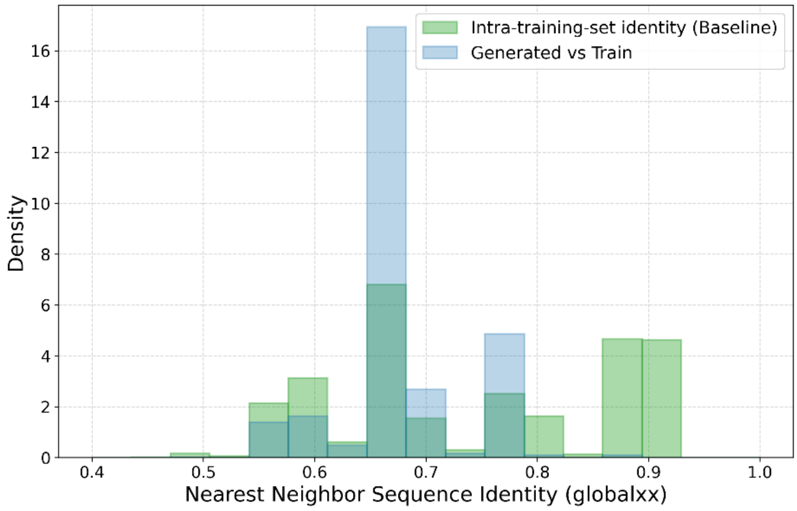

Figure S7. Distributions of nearest neighbor sequence identity for generated peptides against the training set compared to the internal training set baseline. The y-axis shows probability density normalized to unit total area, so the absolute values are arbitrary and meaningful only in shape comparison.

Furthermore, to verify the topological separation between generated and training sequences, we sampled 2,000 generated and 2,000 training peptides and performed hierarchical clustering across multiple identity thresholds (from 50% to 90%). As summarized in Table S8, at stringent thresholds (⩾80%), the mixed cluster rate approaches zero (e.g., 0.03% at 80%), indicating distinct topological segregation without memorization. When the threshold is relaxed to 50% (allowing roughly four amino acid matches in a 9-mer), the peptides merge into broader super-clusters, reflecting the presence of shared anchor motifs necessary for MHC binding. However, under this relaxed condition, the average cluster purity remains remarkably high at 84.52%. This demonstrates that while the model successfully learns required canonical motifs, it synthesizes the non-anchor residues in a novel manner, causing generated peptides to form distinct sub-families within broader motif bounds rather than evenly overlapping with the training data.

Table S8. Clustering topological purity across multiple sequence identity thresholds.

Identity threshold	Total clusters	Pure gen clusters	Pure train clusters	Mixed cluster	Mixed (%)	Avg Purity
90%	3943	2000	1943	0	0.00%	100.00%
80%	3783	1966	1816	1	0.03%	99.98%
70%	3218	1506	1650	62	1.93%	98.45%
60%	2064	603	1148	313	15.16%	91.70%

50%	1005	92	505	408	40.60%	84.52%

References

1. Lin, Z. *et al.* Evolutionary-scale prediction of atomic-level protein structure with a language model. *Science* **379**, 1123–1130 (2023).

2. Song, Y. *et al.* Score-Based Generative Modeling through Stochastic Differential Equations. Preprint at https://doi.org/10.48550/arXiv.2011.13456 (2021).

3. Vita, R. *et al.* The immune epitope database (IEDB): 2018 update. *Nucleic Acids Res.* **47**, D339–D343 (2019).

4. Zhang, Y. *et al.* Epitope-anchored contrastive transfer learning for paired CD8+ T cell receptor–antigen recognition. *Nat. Mach. Intell.* **6**, 1344–1358 (2024).

5. Lu, T. *et al.* Deep learning-based prediction of the T cell receptor–antigen binding specificity. *Nat. Mach. Intell.* **3**, 864–875 (2021).

6. 10x Genomics. A New Way of Exploring Immunity – Linking Highly Multiplexed Antigen Recognition to Immune Repertoire and Phenotype. (2022).

7. Albert, B. A. *et al.* Deep neural networks predict class I major histocompatibility complex epitope presentation and transfer learn neoepitope immunogenicity. *Nat. Mach. Intell.* **5**, 861–872 (2023).

8. 10x Genomics. Cell Ranger. (2025).

9. Islam, R. & Moushi, O. M. Gpt-4o: The cutting-edge advancement in multimodal llm. *Authorea Prepr.* (2024).

10. Liu, A. *et al.* Deepseek-v3 technical report. *ArXiv Prepr. ArXiv241219437* (2024).

11. Team, G. *et al.* Gemini: a family of highly capable multimodal models. *ArXiv Prepr. ArXiv231211805* (2023).

12. Ma, M., Tu, W., Vasquez-Rios, C. & Ding, J. Generating cognate epitope sequences of T-cell receptors with a generative transformer. *bioRxiv* 2025–01 (2025).

13. Chen, Z. *et al.* Binding peptide generation for MHC Class I proteins with deep reinforcement learning. *Bioinformatics* **39**, btad055 (2023).

14. Tang, S., Zhang, Y. & Chatterjee, P. PepTune: De Novo Generation of Therapeutic Peptides with Multi-Objective-Guided Discrete Diffusion. Preprint at https://doi.org/10.48550/arXiv.2412.17780 (2025).

15. Chen, T., Zhang, Y., Tang, S. & Chatterjee, P. Multi-Objective-Guided Discrete Flow Matching for Controllable Biological Sequence Design. Preprint at https://doi.org/10.48550/arXiv.2505.07086 (2025).

16. Chen, L. T. *et al.* Target sequence-conditioned design of peptide binders using masked language modeling. *Nat. Biotechnol.* 1–9 (2025).

17. Wang, X. *et al.* Diffusion language models are versatile protein learners. *ArXiv Prepr. ArXiv240218567* (2024).

18. Cock, P. J. *et al.* Biopython: freely available Python tools for computational molecular biology and bioinformatics. *Bioinformatics* **25**, 1422 (2009).

19. Varga, J. K., Ovchinnikov, S. & Schueler-Furman, O. actifpTM: a refined confidence metric of AlphaFold2 predictions involving flexible regions. *Bioinformatics* **41**, btaf107 (2025).

20. Wu, F. *et al.* Fast and accurate modeling of TCR-peptide-MHC complexes using tFold-TCR. *bioRxiv* 2025–01 (2025).

21. O'Donnell, T. J., Rubinsteyn, A. & Laserson, U. MHCflurry 2.0: improved pan-allele prediction of MHC class I-presented peptides by incorporating antigen processing. *Cell Syst.* **11**, 42–48 (2020).

22. Reynisson, B., Alvarez, B., Paul, S., Peters, B. & Nielsen, M. NetMHCpan-4.1 and NetMHCIIpan-4.0: improved predictions of MHC antigen presentation by concurrent motif deconvolution and integration of MS MHC eluted ligand data. *Nucleic Acids Res.* **48**, W449–W454 (2020).
